# Negative expressions are shared more on Twitter for public figures than for ordinary users

**DOI:** 10.1093/pnasnexus/pgad219

**Published:** 2023-07-06

**Authors:** Jonas P Schöne, David Garcia, Brian Parkinson, Amit Goldenberg

**Affiliations:** Department of Experimental Psychology, University of Oxford, Oxford, Oxfordshire OX2 6NW, UK; Harvard Business School, Harvard University, Boston, Massachusetts 02163, USA; Digital, Data and Design Institute at Harvard, Allston, Massachusetts 02134, USA; Department of Politics and Public Administration, University of Konstanz, Konstanz, Baden-Württemberg 78464, Germany; Department of Psychology, Harvard University, Cambridge, Massachusetts 02138, USA; Department of Computer Science and Biomedical Engineering, Graz University of Technology, Graz, Styria 8010, Austria; Department of Experimental Psychology, University of Oxford, Oxford, Oxfordshire OX2 6NW, UK; Harvard Business School, Harvard University, Boston, Massachusetts 02163, USA; Digital, Data and Design Institute at Harvard, Allston, Massachusetts 02134, USA; Complexity Science Hub Vienna, Vienna, Vienna 1080, Austria

**Keywords:** emotion sharing, social media, Twitter, emotion contagion, public figures

## Abstract

Social media users tend to produce content that contains more positive than negative emotional language. However, negative emotional language is more likely to be shared. To understand why, research has thus far focused on psychological processes associated with tweets' content. In the current study, we investigate if the content producer influences the extent to which their negative content is shared. More specifically, we focus on a group of users that are central to the diffusion of content on social media—public figures. We found that an increase in negativity was associated with a stronger increase in sharing for public figures compared to ordinary users. This effect was explained by two user characteristics, the number of followers and thus the strength of ties and the proportion of political tweets. The results shed light on whose negativity is most viral, allowing future research to develop interventions aimed at mitigating overexposure to negative content.

**Significance Statement**

Though most original social media content expresses positive emotions, content expressing negativity is shared more, inflating the platform's total negativity. In this study, we asked: whose negative content is more likely to be shared. Based on previous research, we suspected public figures' negativity is shared more due to the structure of their social network structure and their produced content. We found that negativity boosts the likelihood of public figures' content being shared, more than ordinary users. This is explained by the fact that negativity is more likely to be shared for weaker social ties and political content. Our findings offer insights into how negativity is shared and provide the basis for interventions reducing overexposure to negativity.

## Introduction

Most original content on social media is positive in affective tone (1–3). Yet, there is a growing realization that negative content is shared more than positive content (4–6). Users' increased tendency to share negative emotions inflates exposure to negativity on social media compared to its true proportion in content production. Overexposure to negativity is known to have adverse consequences at the individual level, leading to a reduction in well-being (7–9). At the collective level, exposure to negativity contributes to group polarization and intergroup conflicts (10, 11). Therefore, it is crucial to understand the roots of negativity sharing online and its driving mechanisms.

Previous research on negativity sharing has mainly focused on specific content-level features and psychological mechanisms that encourage the sharing of negative tweets (5, 12, 13). Here, we hope to tackle the question in a complementary way by asking: *whose* negative content is more likely to be shared? More specifically, we hope to examine whether the association between negativity and sharing is stronger for public figures compared to ordinary users. We hypothesize that such association is stronger for public figures because they have distinctive characteristics that make their negative content more likely to be shared. Specifically, we show that two unique attributes seem to be responsible for the difference between public and ordinary users in the association between negativity and sharing. The first is the fact that public users have weaker ties, who are more likely to share negative emotions (14). The second is that public figures are more likely to write about politics, which is content that is typically more negative and more conducive to sharing (6).

To examine these hypotheses, we first replicated the previous finding showing that original content on social media tends to be more positive. Second, we assessed whether the association between negativity and the number of retweets was stronger for public figures compared to ordinary users. We further examined if this effect was driven by a certain type of public figures. We then compared two user characteristics, number of followers and proportion of political tweets, and assessed which of these user characteristics were associated with an increased likelihood that the negative content users generated was shared. Finally, we tested whether the differential effect of negativity on sharing for public figures and ordinary users was mediated by their distinctive user characteristics.

### Negativity sharing on social media

Shared content represents up to 75% of all content that people see on social media (15). It is therefore important to understand what context is more likely to be shared. Generally speaking, language that contains more emotional content is more likely to be shared (16), but one central question is whether positive or negative emotional language leads to more sharing. Although some studies have suggested that positive content, such as scientific articles (17), Olympic Games posts (18), or news articles via email (19), is shared more than negative content, other studies have found that negative content tends to be shared more frequently than positive content in other contexts (4–6). The tendency to share negative content can be found in different cultures and platforms, including Facebook (5), Twitter (20), and Weibo (14).

Previous research has suggested several reasons why negativity might be shared more than positivity. The first reason is that heightened attention to negative content, also known as the negativity bias (21–23), may lead to more engagement and sharing (13, 24). The impact of the negativity bias seems to be moderated by tie strength (25), with negativity shared more between weaker ties, while positivity is shared more between close ties (14, 26). Given that negativity is more likely to be shared between weaker ties (25, 26), negativity should be more viral for users with a higher proportion of weak ties, such as public figures. A second reason why negativity is more likely to be shared is specific to political and intergroup discourse, which is frequent on social media (27–29). Users who write political tweets are often driven by intergroup hostility and reputation considerations, which might lead them to share more negative content (5, 6, 30). Therefore, users who are writing about politics more often may be more likely to have their negative content shared. It is important to note, however, that attention to political content and negativity sharing may also be driven by a bias in the literature toward political figures and news media. Recent research suggests that despite the fact that Twitter users seem to be more engaged with politics than the average US population (31), the majority of users (60%) do not follow any political public figures on Twitter (32).

### Public figures and ordinary users

Verification status is one of the distinguishing features between users on major social media websites such as Twitter, Facebook, and Instagram. Verified users encompassed a wide range of public figures, including politicians, journalists, celebrities, and athletes. At the time of our data collection, the verification status on Twitter indicated whether a user was a public figure authenticated by the platform or not. The verified status of users changed on 2022 November 5, after which every user was able to verify their account for $8 a month. Before the transition, verified users made up only a small proportion of all users. For example, Twitter has 229 million active users, of which only 420,300 (0.18%) were verified. Despite their relatively small number, verified users are central to the diffusion of content online (33, 34).

Public figures have distinctive characteristics on social media, which may affect the extent to which their negativity would be associated with sharing. The first characteristic is their *high number of followers* (35), which often means that many of these ties are weak ties (36). Given that weaker ties are more likely to share negative content than positive content (14, 25), negative content generated by users who have many followers, such as public figures, is more likely to be shared compared to other types of content. The second characteristic of public figures on social media is that they produce a relatively *higher proportion of political content*. Public figures use social media not only to promote themselves but also to promote social and political causes (37–39). Additionally, many verified users on social media are political figures, journalists, or other users who specialize in politics, making them more likely to produce political content. Given that negativity is especially likely to be shared in political content (5, 6, 20), negative content from users who produce a higher proportion of political content is more likely to be shared.

Previous research has already demonstrated a positive link between negativity and increased content sharing when focusing on public figures such as political leaders or news outlets (20, 40, 41). However, these previous studies have not compared public figures to ordinary users and have not examined for whom the association between negative emotions and sharing is stronger. This question seems to be crucial if the ultimate goal is to find ways to reduce negativity sharing on social media. Furthermore, given that research on public figures has mostly focused on news media and political figures, it remains unclear whether the observed relationship between negativity and content diffusion can be generalized beyond this specific subset of public figures.

### The present research

The primary goal of this study was to assess whether the association between negativity and content sharing is stronger when the content is produced by verified users compared to when it is produced by ordinary users. We further examined whether specific user characteristics—number of followers and proportion of political tweets—can account for this difference in the strength of association. To achieve these goals, we first tested whether the type of user (ordinary user, public figure) moderated the association between negative content and sharing. As there are various types of public figures who can obtain verification status, we further tested if certain types of public figures were more likely to be differentiated from ordinary users in the association between negative language and sharing. We then verified that public figures have relatively more followers and produce a higher proportion of political tweets before investigating if these characteristics mediated the differential effect of expressed sentiment on sharing of content produced by public figures and ordinary users. The analyses were not preregistered, but the data and code are available at https://osf.io/xuraq/.

## Results

Using the Twitter Application Programming Interface (API), we first compiled a list of users (*n* = 45,918) and their account descriptions and then extracted their tweets in 2019 January (see *Materials and methods* for more information on our data extraction procedure). We classified users into two groups, public figures and ordinary users, based on account verification status. At the time of our data collection, public figures had a blue checkmark indicating that their account was verified by Twitter, while ordinary users were not verified. Triangulating a few classification methods, we further classified public figures into categories, including entertainment, journalists, news outlets, organizations, politics, sports, and others. For a detailed description and breakdown of this classification, see *Materials and methods* and Section S5 and Tables S10–S12. We then selected an equal number of public figures and ordinary users (*n* = 6,678) who were matched by their activity level on the platform using propensity score matching. This method involved matching ordinary users to public figures based on their tweet count, as described in detail in *Materials and methods*, resulting in a total sample of 427,502 tweets from public figures and 428,213 tweets from ordinary users (see *Materials and methods* and Section S1, Tables S1–S3, and Figs. S1–S5 for analysis using the full sample). To assess user characteristics, we collected data on each user's number of followers as a measure of tie strength and analyzed their proportion of political tweets.

Following our process of user identification, we then turned to processing the tweets produced by the users. A retweet occurs when one user shares another user's message with his or her own social network (42). For each tweet, we retrieved the number of retweets and evaluated the affective content of each tweet using the preevaluated sentiment analysis tool VADER (43). For each tweet, VADER generates a continuous sentiment score ranging from −1 (extremely negative) to +1 (extremely positive), along with an overall valence categorization (positive, neutral, and negative). A tweet is classified as positive if the sentiment score exceeds 0.2, negative if it falls below −0.2, and neutral if the score falls between the two values. See *Materials and methods* for a detailed explanation of the tweet evaluation process. We further compared the results of different sentiment analysis tools in Section S2, Table S4, and Fig. S6. We identified political tweets using Latent Dirichlet Allocation (LDA) topic modeling (44), which uses the co-occurrence of words or phrases to identify a predefined number of underlying themes. This method is capable of identifying political tweets because political content often contains similar words, such as the names of political figures or events. To identify the topic that represents political tweets, we manually inspected the most frequently occurring words in each topic and selected the one that contained political terms. More details on the assessment of these characteristics and their transformations in *Materials and methods* and Section S3, Tables S5–S9, and Figs. S7 and S8 for details on the different configurations of topic modeling.

### Frequency of positive, negative, and neutral content for public figures and ordinary users

We tested if public figures and ordinary users produced more positive compared to negative and neutral affective content using the three VADER categories. For this analysis, we counted the number of a user’s tweets in each of VADER's three affective categories. As we matched both user types by their total number of tweets, we were able to compare the absolute number of tweets in the given categories as the dependent variable. Using linear regression models, we predicted the total number of tweets per affective category based on user type (public figure and ordinary user).

**Fig. 1.**
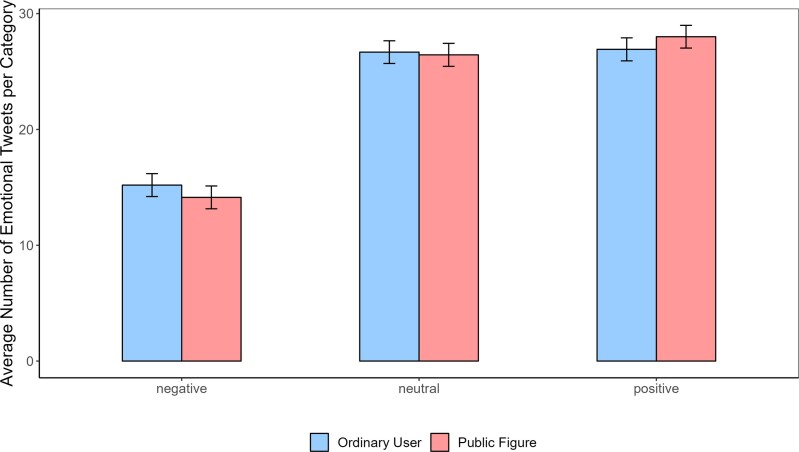
Number of affective tweets by categories for both user types. The bar graphs show that for both public figures and ordinary users, negative affective content is the least frequent type of originally created content, replicating previous findings. Additionally, public figures seem to produce even less negative content compared to ordinary users.

As expected, positive affective content was more frequent than negative for both user types (*b* = −12.79 [−13.78, −11.80], SE = 0.50, *t* (37,358) = −25.40, *P* < 0.001, *R*^2^ = 0.021; see Fig. 1).^1^ The difference between positive and neutral content was only marginally significant (*b* = −0.90 [−1.88, 0.083], SE = 0.50, *t* (37,358) = −1.79, *P* = 0.072, *R*^2^ = 0.021). Looking at the comparison between public figures and ordinary users, we found that public figures produced a similar amount of positive content compared to ordinary users (*b* = 1.09 [−0.30, 2.48], SE = 0.71, *t* (37,358) = 1.53, *P* = 0.12, *R*^2^ = 0.021), a similar amount of neutral content (*b* = −1.32 [−3.29, 0.64], SE = 1.01, *t* (37,358) = −1.31, *P* = 0.18, *R*^2^ = 0.021), but most importantly less negative content compared to ordinary users (*b* = −2.15 [−4.12, −0.17], SE = 1.01, *t* (37,358) = −2.13, *P* = 0.032, *R*^2^ = 0.021). These results suggest that public figures tended to produce fewer negative tweets compared to ordinary users.

### Associations between sentiment scores and the number of retweets for public figures and ordinary users

To examine the association between negativity sharing and retweets for each user type, we conducted an interaction between the user type (public figure and ordinary user) and the continuous sentiment score from VADER in predicting the log-modulus-transformed number of retweets (see *Materials and methods* for more details on the transformation). According to previous research, an increase in both positive sentiment and negative sentiment should lead to more retweets; therefore, we decided to fit a quadratic mixed model to predict the number of retweets using a quadratic function of the continuous sentiment score between −1 and 1. We also investigated potential nonquadratic relationships between sentiment and sharing, but the quadratic model seemed to produce the most predictive model (see Section S7, Tables S14 and S15, and Figs. S12 and S13). A quadratic function (*y* = *ax*^2^ + *bx* + *c*) returns three coefficients describing the parabola. The coefficient *a* defines how wide the U-shaped graph is and if it opens upward or downward. If *a* is positive, then the parabola opens upward, and if *a* has a higher absolute value, this means that the line slopes more steeply. The coefficient *b* represents whether and to what extent the local peak is a positive or a negative *x* value. If *b* is negative, the local peak is a positive *x* value, and if *b* is positive, the local peak is a negative *x* value. The coefficient *c* is the intercept with the *y*-axis (at *x* = 0). A larger *a* coefficient in the present model would indicate a stronger association between sentiment and content sharing, while a larger *b* coefficient would suggest that positivity was shared more than negativity. In other words, the coefficient *a* informs us about the overall influence of emotional intensity or extremity on content sharing, while the coefficient *b* informs us about whether positivity or negativity is more likely to be retweeted. To account for differences in the number of tweets produced by users in our model, we included a random intercept for each user.

**Fig. 2.**
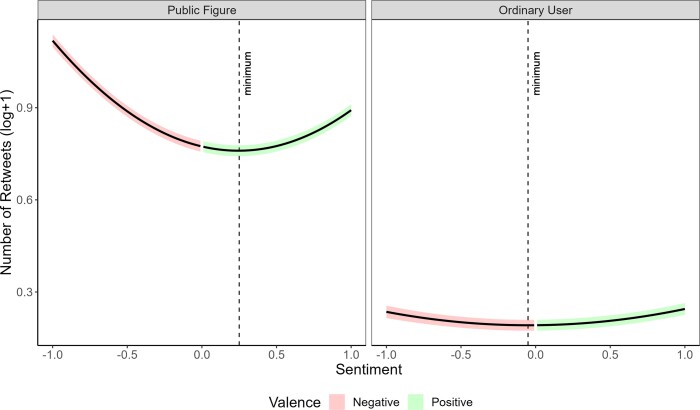
Number of retweets as a function of sentiment and user type. The results suggest that stronger sentiment is associated with more retweets for both types of users. The local minimum for public figures is reached with a more positive emotional tweet, indicating that negativity is more strongly positively associated with the number of retweets for public figures than for ordinary users. Public figures also received more retweets for neutral content than ordinary users.

Looking first at the interaction, results suggested that higher sentiment values (positive or negative) were more strongly positively associated with the number of retweets for content produced by public figures than for content produced by ordinary users (*a* = 0.18 [0.17, 0.19], SE = 0.0067, *t* (845,141.16) = 27.17, *P* < 0.001, *R*^2^ = 0.085; see Fig. 2). This means that emotional content in general was more likely to be shared for public figures. The extent to which negativity led to more content sharing than positivity was also greater for public figures than for ordinary users (*b* = −0.12 [−0.12, −0.10], SE = 0.0036, *t* (845,324.79) = −31.95, *P* < 0.001, *R*^2^ = 0.085).

Having established the fact that the association between negativity and sharing was stronger for public figures, we then examined whether this effect was driven by certain types of public figures. To achieve this, we replicated the previous mixed model analysis, using the log-modulus-transformed retweet count as our outcome variable and the interaction of two predictors: the quadratic function of the continuous sentiment score from VADER and the user type category. Unlike our previous analysis, where the user type variable was a simple binary variable (verified users vs. ordinary users), we expanded it to include multiple user types, including ordinary users and seven types of public figures. We also included a random intercept for individual users.

We found that negativity was shared more for all types of public figures compared to ordinary users (see Section S5, Table S12, and Fig. S9 for full description), even in comparison to the subset of public figures for whom negativity was associated with the smallest increase in retweets, namely, organizations (*b* = −0.034 [−0.044, −0.023], SE = 0.0053, *t* (845,324.79) = −6.37, *P* < 0.001, *R*^2^ = 0.053). Negativity increased retweets most for political figures (*b* = −0.32 [−0.35, −0.30], SE = 0.012, *t* (845,324.79) = −26.51, *P* < 0.001, *R*^2^ = 0.053), followed by news outlets (*b* = −0.21 [−0.24, −0.16], SE = 0.020, *t* (845,324.79) = −10.19, *P* < 0.001, *R*^2^ = 0.053).

### Differences in user characteristics between public figures and ordinary users

We suspected that two variables can explain why negativity is more frequently shared for public figures: the difference in the number of followers and the proportion of political tweets. We first needed to establish that public figures indeed have a higher number of followers and a greater proportion of political tweets than ordinary users. We used a simple linear regression with a dummy-coded variable for verification status to predict the log-modulus-transformed number of followers and the proportion of their political tweets. The proportion of political tweets was defined as the number of political tweets identified by topic modeling divided by the total number of tweets.

**Fig. 3.**
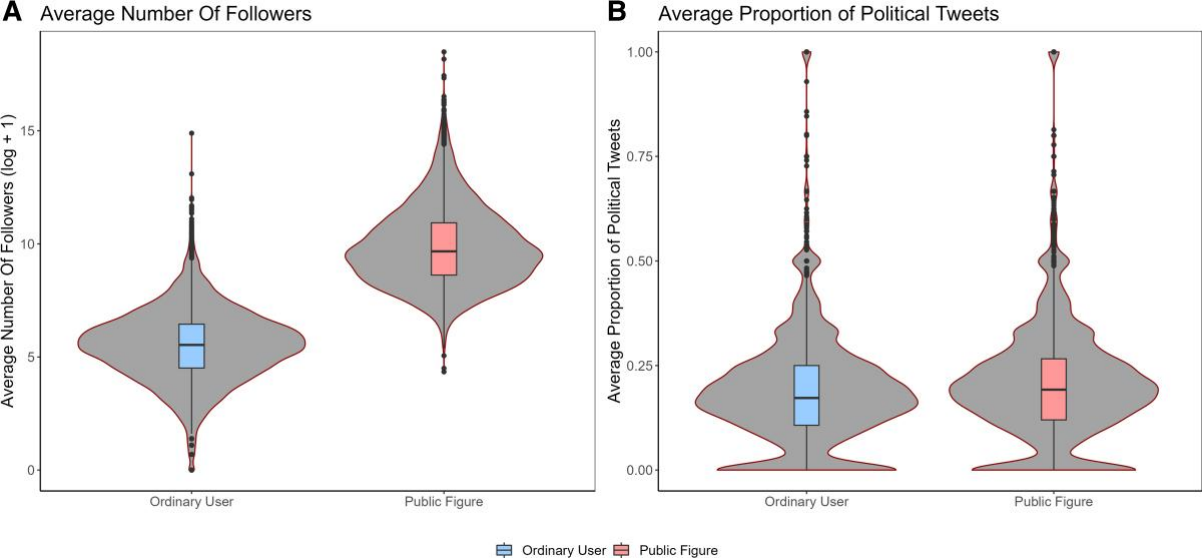
Differences between public figures and ordinary users. Results suggest that public figures have more followers than ordinary users A) and produce a higher proportion of political tweets B).

Compared to ordinary users, public figures had more followers (*b* = 4.40 [4.34, 4.46], SE = 0.029, *t* (13,352) = 151.3, *P* < 0.001, *R*^2^ = 0.63; see Fig. 3a) and produced approximately 2% more political content (*b* = 0.019 [0.014, 0.024], SE = 0.0025, *t* (13,352) = 7.48, *P* < 0.001, *R*^2^ = 0.041; see Fig. 3b). While the proportion of political tweets only slightly differed between verified and ordinary users (around 2% increase), the difference in their number of followers was more substantial (*d* = 0.01 vs. *d* = 0.16). This suggests that the follower count is likely a more salient factor in differentiating public figures from nonpublic figures than political tweet content.

### Association between user characteristics and negativity sharing

**Fig. 4.**
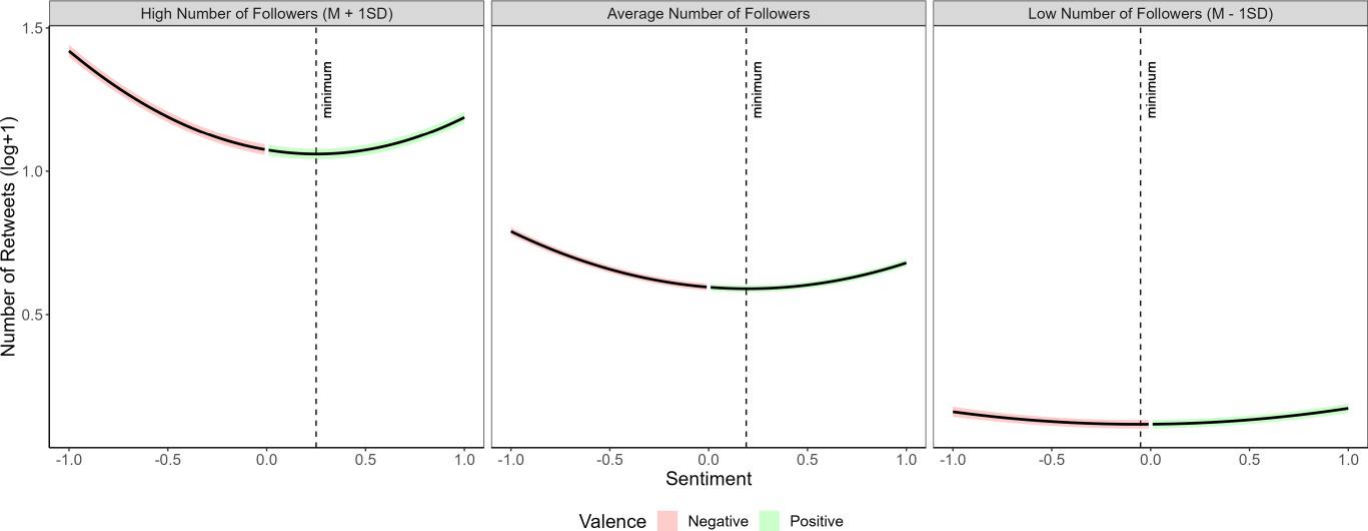
Number of retweets as a function of sentiment and number of followers. To visualize the interaction between two continuous variables (sentiment and the number of followers), the panels show the mean number of followers in the middle panel as well as the *M* ± SD (left and right panels, respectively). The colored areas indicate the 95% confidence intervals. The results show that the effect of sentiment on content sharing is greater when there is a higher number of followers. In other words, negativity sharing was stronger for the content of users with more followers.

After confirming that public figures had more followers and talked more about politics, we tested if these characteristics moderated the effect of sentiment on the number of retweets. To achieve this, we conducted two mixed models with quadratic terms. First, we looked at the interaction between the quadratic term of the continuous sentiment score and the number of followers in predicting the number of retweets. We also included a random intercept for users in both models. We found a stronger association between general sentiment and the number of retweets (*a* = 0.049 [0.083, 0.096], SE = 0.0033, *t* (846,247.48) = 26.88, *P* < 0.001, *R*^2^ = 0.21; see Fig. 4) and more specifically between negativity and the number of retweets (*b* = −0.061 [−0.064, −0.057], SE = 0.0018, *t* (846,530.08) = −33.29, *P* < 0.001, *R*^2^ = 0.21) for tweets produced by users with more followers. Given the dramatically higher number of followers of verified users, we wanted to make sure that the effect of the number of followers is not limited to verified users. We, therefore, repeated this analysis using only the subsample of ordinary users, finding a similar effect (see Section S4). Additionally, we tested another model in which we matched a subset of ordinary users and public figures based on their number of followers. We found that negativity was shared more for ordinary users that have as many followers as some public figures, although not to the same extent (see Section S4 for detailed discussion).

**Fig. 5.**
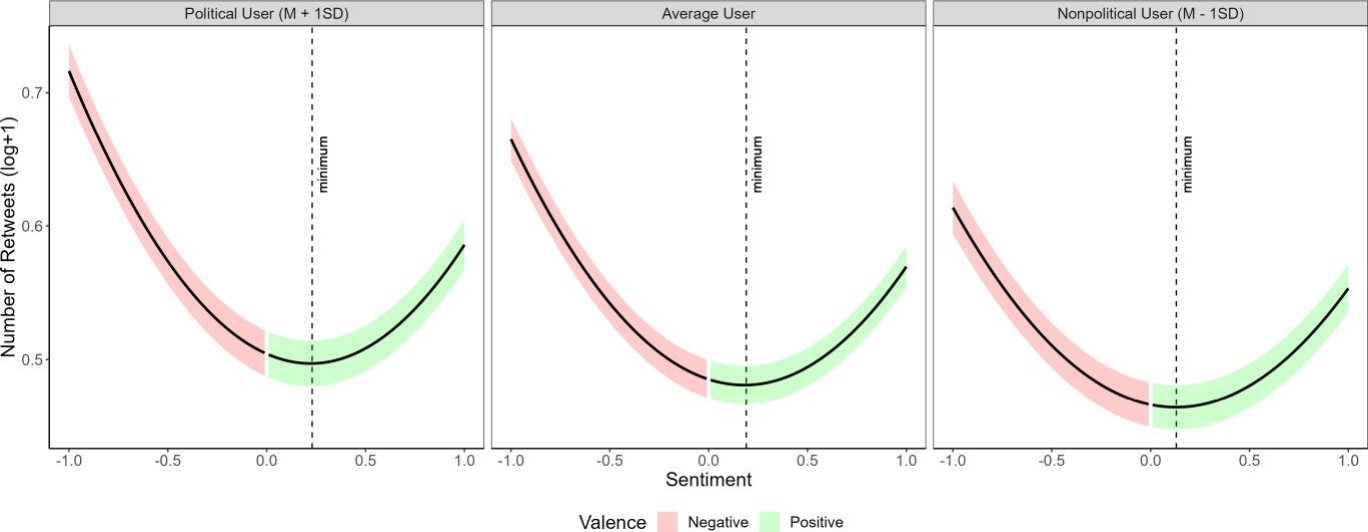
Number of retweets as a function of emotionality and proportion of political tweets. To visualize the interaction of two continuous variables (sentiment and the proportion of political tweets), the panels show the predicted association between sentiment and the number of retweets at the mean proportion of political tweets in the middle panel as well as the *M* ± SD (left and right panels, respectively). The colored areas indicate the 95% confidence intervals. The results indicate that tweets produced by users with a higher proportion of political tweets show stronger associations between sentiment and content sharing and between negativity and content sharing.

In the second model, we examined the interaction between the quadratic term of the continuous sentiment score and the proportion of political tweets. We again used a random intercept for users as in the previous models. We found that tweets that were produced by users with a higher proportion of political tweets showed a stronger association between general sentiment (positive or negative) and the number of retweets (*a* = 0.014 [0.0078, 0.021], SE = 0.0034, *t* (854,258.36) = 4.23, *P* < 0.001, *R*^2^ = 0.014; see Fig. 5) and between negativity and the number of retweets (*b* = −0.017 [−0.021, −0.013], SE = 0.0019, *t* (854,302.00) = −9.17, *P* < 0.001, *R*^2^ = 0.014).

### Parallel mediation analysis

We hypothesized that negative content produced by public figures was shared more due to the differences in user characteristics that promote negativity sharing. To assess this prediction, we conducted a parallel mediation analysis assessing two potential mediators of the effects of user type (public figures vs. ordinary users) on sharing of their negative content (47). To conduct the mediation, we needed an individual-level variable that reflected the degree to which negativity was shared for that user. We computed a new dependent variable that quantified the extent to which an increase in negativity was associated with more retweets for every individual user in our dataset, which allowed us to predict how much negativity is shared for certain users depending on their characteristics. This negativity sharing–dependent variable was calculated using a similar model to the previous ones with two additional changes. First, instead of a quadratic mixed model, we used a split regression to approximate the U-shaped relationship between sentiment and the number of retweets. A split regression contains a categorical variable that is inserted into a linear regression model as an interaction factor to allow for separate slopes for different categories. In our case, we split the continuous variable sentiment using a binary categorical variable into values <0 (negative slope) and ≥0 (positive slope). This approach allowed us to derive a single coefficient specifically representing the association between an increase in negativity and the number of retweets. Second, we introduced random slopes representing the relationship between sentiment and the number of retweets for each user. Another benefit of using a split regression for the extraction of a per-person coefficient was that a linear regression uses one coefficient to describe the relationship between sentiment and retweets (the beta coefficient), while a quadratic regression uses two (coefficients *a* and *b*, describing how much sentiment was associated with retweets as well as if negative emotions were shared more than positive emotions). We extracted the random slope describing the extent to which an increase in negative emotion was associated with the number of retweets as our dependent variable for the mediation analysis. The potential mediators were the two user characteristics identified above, namely, the number of followers and the proportion of political tweets. We used the PROCESS v4 macro for RStudio by Hayes (47) to conduct the parallel mediation analysis.

**Fig. 6.**
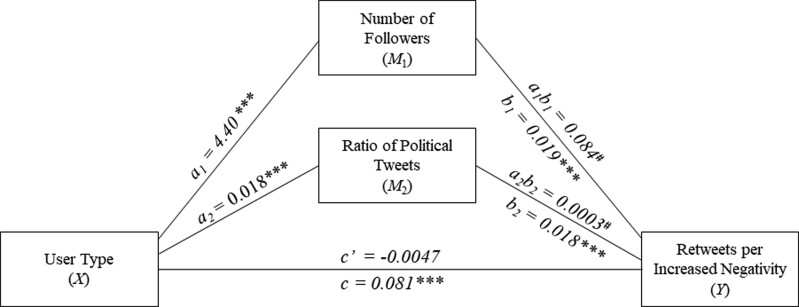
Parallel mediation analyzes the effect of user type on negativity sharing via two different user characteristics. User type positively predicts user characteristics: number of followers and proportion of political tweets. The indirect effects of user type on negativity sharing via the number of followers and the proportion of political tweets were both significant.

Starting with the *a*-paths in our parallel mediation model, the user type was, as reported above, a significant positive predictor of both the number of followers (*a*_1_ = 4.40 [4.33, 4.45], SE = 0.029, *t* (13,352) = 150.82, *P* < 0.001, *R*^2^ = 0.63; see Fig. 6) and the proportion of political tweets (*a*_2_ = 0.019 [0.014, 0.024], SE = 0.0025, *t* (13,352) = 7.47, *P* < 0.001, *R*^2^ = 0.0042). Both the number of followers (*b*_1_ = 0.019 [0.018, 0.0021], SE = 0.0006, *t* (13,352) = 31.01, *P* < 0.001, *R*^2^ = 0.13) and the proportion of political tweets were also significant predictors of sharing of negative content (*b*_2_ = 0.018 [0.043, 0.0032], SE = 0.0072, *t* (13,352) = 2.55, *P* = 0.01, *R*^2^ = 0.13). While the total effect of user type on negativity sharing was significant (*c* = 0.081 [0.076, 0.085], SE = 0.0022, *t* (13,352) = 37.08, *P* < 0.001, *R*^2^ = 0.13), the direct effect controlling for the mediators rendered this effect nonsignificant (*c*′ = −0.0047 [−0.011, 0.0021], SE = 0.0035, *t* (13,352) = −1.36, *P* = 0.17, *R*^2^ = 0.13). A 95% bias-corrected confidence interval based on 10,000 bootstrap samples indicated that the sampled indirect effects of user type on sharing of negative tweets via the number of followers (*a*_1_*b*_1_ = 0.083, SE = 0.0039), while holding the other mediator constant, were consistently above zero (0.075–0.091). These findings indicate a significant positive indirect effect, suggesting that the number of followers mediates the relationship between the user type and the sharing of negative tweets. Similarly, the sampled indirect effects via the proportion of political tweets were also consistently above zero (*a*_2_*b*_2_ = 0.0004 [0.0001–0.0006], SE = 0.0010). These results from the parallel mediation analysis suggest that the user type only had an indirect effect on negativity sharing. This effect was mediated by both the user's number of followers and proportion of political tweets.

## Discussion

In this project, we compared the extent to which emotional content is shared for public figures and ordinary users. We found that despite the fact that public figures tended to produce less negative content than other users, the association between the increase in emotional intensity, especially negativity, and the number of retweets the post received was stronger for public figures compared to ordinary users. This stronger association between negativity and sharing was consistent among all types of public figures, while we did not find that negativity was shared more than positivity for ordinary users. We identified two user characteristics—the number of followers and the proportion of political content—that mediated the effect of user type on the extent to which negativity was associated with an increase in retweets. When comparing these two mechanisms, it seemed that the number of followers was a stronger mediator to the differences between user types. This work supplements previous research on sharing of negativity, which has mostly focused on psychological processes elicited by negative emotions in tweets (4, 6, 20).

**Fig. 7.**
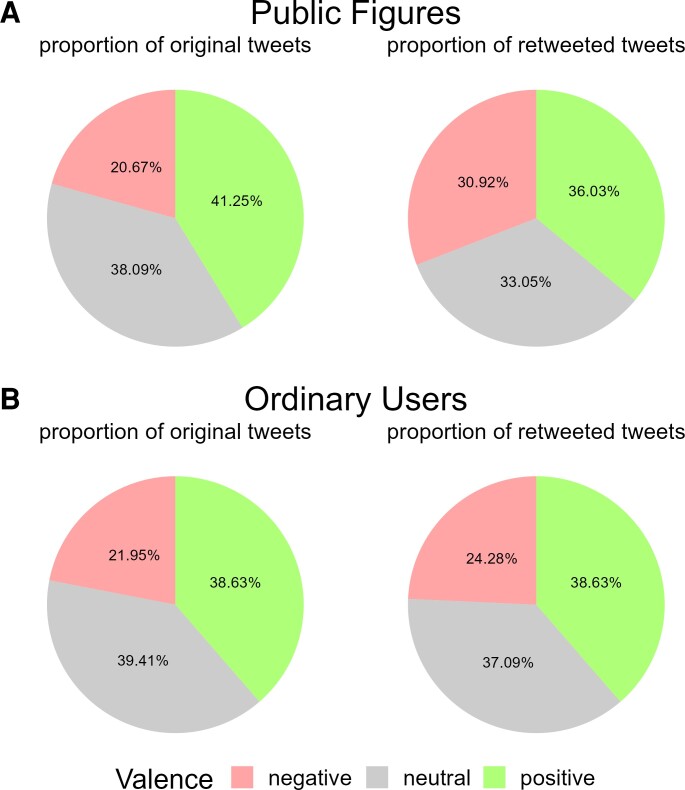
Relative frequencies of emotional content in original tweets and retweets of public figures A) compared to ordinary users B). Although negative content only made up 20.67% of the original tweets for public figures, it accounted for 30.92% of all retweets, signifying an increase of 10.25%. Conversely, for ordinary users, the proportion of negative content increased only slightly from 21.95 to 24.28% (2.33%). In contrast, when examining content that is less likely to be shared, such as neutral content for both user types, the proportion of such content decreases. Consequently, this content is underrepresented in retweets in comparison to its original frequency. These results suggest that the virality of negative content for public figures can lead to an inflation of their content compared to the original texts they produce.

Public figures seem to contribute substantially to people's exposure to negative content on social media. Whenever a tweet is shared, it is duplicated and displayed to the sharer's followers. Considering the fact that public figures have a much larger number of followers and given their centrality in social media networks (33, 34), their shared content makes up a large share of the material presented on social media (see Fig. 7). The resulting negatively biased sample of retweeted content then may lead other users to infer that the most credible and popular users on social media platforms use negative language, which in turn might negatively influence emotion expression norms.

Disproportionate sharing of public figures' negative content could have adverse implications for both individuals and collectives on social media. Overrepresentation of negative information, such as negative news or online hate, cultivates a more negative evaluation of the world (48), potentially leading to a decrease in social trust (49) and a reduction in subjective well-being (50, 51). In addition, exposure to negative political content has negative collective consequences such as contributing to group polarization and intergroup conflicts (10, 11). This study's findings also provide an explanation for why the overrepresentation of negative emotional content produced by public figures has worsened over time (52). The increased sharing of negative affective content incentivizes public figures to generate more of it (53), thereby perpetuating the cycle of negativity on social media.

### Limitations and future directions

While this work provides new insights into how negativity is shared online and despite our efforts to address alternative hypotheses, the current analysis has limitations that should be addressed in future work. The most important limitation is the observational nature of this study, which means that we could not manipulate user characteristics while controlling for others that differed between public figures and ordinary users such as average emotions expressed (see Section S6, Tables S13, and Figs. S10 and S11 for the influence of average emotions expressed on negativity sharing). In future studies, researchers should manipulate user characteristics by using a curated news feed look-alike that allows for the manipulation of such user characteristics.

The second limitation pertains to our assessment of user characteristics. While it is plausible that the average tie strength decreases as the number of followers a user has increased, we do not have a direct measure for tie strength, such as reciprocal connections or mutual interactions. In a similar vein, political content was classified using topic modeling, which can be implemented in various ways. This raises the question of how accurately this classification can categorize political content. To alleviate some of these concerns, we tested different configurations of the topic modeling classifications, still finding similar results (see Section S3, Tables S5–S9, and Figs. S7 and S8). Future work should sample entire networks over time to measure how much users interact with each other to get a more fine-grained measurement of tie strength as well as a user's general tendency to create political content.

In addition to addressing the abovementioned limitations, future research should seek to develop interventions designed to minimize general overexposure to negative content by targeting the abovementioned user characteristics. We identified users whose negative content had the highest tendency to be shared. As negative content is in fact produced more rarely than positive content (2), an effective intervention should aim to prevent the followers of such users from disproportionally sharing negative content. One possible way of doing this is by educating users about the consequences of sharing negative content of users who have a high number of followers or by providing them with feedback about their tendency to share negative content produced by much-followed users (54). Given that the underlying mechanisms are likely driven by psychological tendencies, the increased consumption of negativity and the associated well-being risks may also manifest in other online contexts, such as browsing behavior (55), as well as in offline contexts, such as news consumption (40). As a result, solely intervening on social media may only address a fraction of the well-being risks associated with Internet use.

We believe that our findings emphasize the crucial role that users with large followings such as public figures have in the dissemination of negative content. Furthermore, the findings shed light on the mechanisms that are involved in the process of sharing negative content and provide the basis for developing interventions aimed to combat the exposure of negative content online.

## Materials and methods

This research adhered to the best practice guidelines for Internet-mediated research set forth by the Central University Research Ethics Committee (CUREC) of the University of Oxford. According to these guidelines, the analysis of public data does not require further ethics approval.

### Participants

Based on previous research, we aimed to collect at least 350,000 tweets per user type to detect the effect of emotion expression on sharing (4, 6). We estimated that in 1 month, we could collect approximately 7,000 ordinary users (assuming that the median number of tweets is 50) (56). To collect a list of users from both user types, we used two separate approaches. For public figures, we first retrieved a full list of all public figures from the @verified Twitter account (*N* = 314,373) and their basic profile statistics. Next, we downloaded their tweets in the period of 2019 January 1 to 2019 January 31. Our final sample included 2,246,068 tweets produced by 39,241 public figures. We then turned to ordinary users. Because there is no suitable method to sample random ordinary users directly, we extracted account names from randomly sampled tweets. We used the 1% Spritzer stream, a real-time stream of a random selection of 1% of all tweets, to collect random tweets between 2019 January 14 and 2019 February 13, which were produced by ordinary users as indicated by the absence of verification status. We then obtained user names from the producer of these random tweets. After discarding duplicated users, we collected the profile statistics such as their number of followers of 6,681 users from this list and retrieved their tweets as well as their descriptive information of these tweets including the number of retweets produced in 2019 January (1,927,684 tweets) using the Twitter API. We removed all tweets that were not in English and nonoriginal tweets, meaning that we removed retweets that did not contain their own added text, resulting in a final sample of 428,223 tweets produced by 6,678 ordinary users.

To achieve an equal sample size of users with similar Twitter activity, we used propensity score matching to match ordinary users to public figures based on their tweet count (57). This statistical technique helps address possible confounding factors in observational studies driven by inherent differences in samples. First, we calculated a propensity score for each user, indicating their likelihood of tweeting during the given month. Then, we employed the nearest neighbor method to match verified and ordinary users with similar propensity scores. This approach ensured that the two groups produced a similar number of tweets during the 1-month period, reducing any potential distortions resulting from different tweeting behaviors. Each ordinary user was matched to one public figure with the closest tweet count. After matching, the sample included 6,678 users of each type, with 427,502 tweets from public figures and 428,213 tweets from ordinary users. We repeated the analysis from *Results* using the full sample of public figures before matching, finding similar results (see Section S1, Tables S1–S3, and Figs. S1–S5).

### Measures

#### Sentiment analysis

We used the sentiment analysis tool VADER (43) to estimate the affective content of tweets. VADER was specifically developed for sentiment analysis in social media and is especially suited for short texts such as those posted on Twitter (58). For each tweet, VADER returns a categorization of the content's overall valence (positive, neutral, and negative) as well as a continuous sentiment score ranging from −1 (extremely negative) to +1 (extremely positive). For the statistical analysis, we used the continuous sentiment score. We repeated the analysis using a different sentiment analysis tool (SentiStrength) (59) (see Section S2, Table S4, and Fig. S6 for more details).

#### Topic modeling

To identify users who produced a higher proportion of political tweets, we first needed to distinguish political tweets from nonpolitical tweets. We used LDA topic modeling to identify political content in tweets (60, 61). LDA clusters texts into a predefined number of topics representing distinct themes. This enabled us to assess the extent to which each sampled user produced political content. We conducted the topic modeling analysis in RStudio (version 4.0.2) using the “topicmodel” package (62).

The specificity/generality of the topics that are identified depends on how many of them are preselected by the investigator. If the investigator decides to examine a small number of topics, topic modeling will use broad brushstrokes to divide the content but ignore finer distinctions. By contrast, specifying a large number of topics can result in topics that are too specific for the particular research question. Choosing the number of predefined clusters is done to balance the specificity and interpretability of the created topics (63). Our goal in the topic number selection was to find one general political topic using the smallest number of topics possible to avoid having multiple, more specific political topics.

The meaning of a topic was assessed qualitatively by analyzing the words used most frequently in this topic (64). The frequency of a word in a topic is expressed in the *β*-score (“beta-scores”). After manual exploration of the semantic coherence of the topics, we found that using five topics created one topic that seemed to be almost exclusively about politics (as indicated by high *β*-scores for words such as “Trump,” “president,” “vote,” “government,” etc.; see Section S3, Tables S5–S9, and Figs. S7 and S8 for all efforts and details of the identified topics). After deciding on the number of topics, we derived *γ*-scores from the LDA analysis, which are percentage estimates of the likelihood that each tweet contained each of the specified topics. Based on this criterion, 25.03% of our sampled tweets were categorized as political tweets, which is similar to previous assessments of the quantity of political content on Twitter (65).

#### User-level variables

Our user-level variables were the user type, number of followers, and proportion of political tweets. A user was categorized as the user type of either public figures or ordinary users depending on whether the account was verified or not. Verification status and the number of followers were extracted from users' basic account information. However, the distribution of the number of followers was skewed and contained zero values. We, therefore, performed a log-modulus transformation (*y* = log(*x* + 1)) on this variable before conducting our statistical analysis. The proportion of political tweets was calculated as the number of the user's tweets that were categorized as political by the topic modeling analysis (as described above) divided by their total number of tweets.

For verified accounts, we further classified them into several major categories of verified users, including political figures, journalists, news outlets, entertainment, sports, and organizations, and evaluated the tendency of their negative content to be shared by other users. To do so, we employed three classification approaches in conjunction to evaluate these categories. Our first approach was to analyze the most frequent words in users' profile descriptions, in order to identify potential categories and build manually curated dictionaries that describe the words used to classify users into their respective types (see Section S5 and Tables S10 and S11 for all categories and corresponding word parts). Second, we matched users based on their identifiers with lists from previous research that had already classified them into specific public figure types. These lists include those created by Barberá (66) as well as Rathje et al. (5) for political figures and the documentation by Bellovary et al. (20) for media outlets. Finally, we employed the tool “Demographer” (67), which utilizes machine learning and natural language processing techniques to infer whether an account belongs to an individual or organization from multilingual social media data.

#### Tweet-level variable

We used the number of retweets as the main dependent variable. Because the distribution of the number of retweets was skewed and contained a high frequency of zeros, we performed a log-modulus transformation before statistical analysis.

## Supplementary Material

pgad219_Supplementary_Data

## Data Availability

The data and code used for this study are available on OSF at https://osf.io/xuraq/.

## Footnotes

1 Marginal *R*^2^ calculated based on recommendations from (45) using R package “MuMIn” (46).

## Preprints

This manuscript was published as a preprint at 10.31234/osf.io/wng5v.

## References

[1] Dodds PS , et al 2015. Human language reveals a universal positivity bias. Proc Natl Acad Sci. 112:2389–2394. 25675475 10.1073/pnas.1411678112 PMC4345622

[2] Ferrara E , Yang Z. 2015. Quantifying the effect of sentiment on information diffusion in social media. PeerJ Comput Sci. 1:e26.

[3] Baylis P , et al 2018. Weather impacts expressed sentiment. PLoS One. 13:e0195750. 10.1371/journal.pone.0195750 PMC5918636 29694424

[4] Brady WJ , Wills JA, Jost JT, Tucker JA, Van Bavel JJ. 2017. Emotion shapes the diffusion of moralized content in social networks. Proc Natl Acad Sci. 114:7313–7318. 28652356 10.1073/pnas.1618923114 PMC5514704

[5] Rathje S , Van Bavel JJ, Van Der Linden S. 2021. Out-group animosity drives engagement on social media. Proc Natl Acad Sci. 118:e2024292118. 10.1073/pnas.2024292118 PMC8256037 34162706

[6] Schöne JP , Parkinson B, Goldenberg A. 2021. Negativity spreads more than positivity on Twitter after both positive and negative political situations. Affect Sci. 2:379–390. 36043036 10.1007/s42761-021-00057-7 PMC9383030

[7] Diener E , et al 2010. New well-being measures: short scales to assess flourishing and positive and negative feelings. Soc Indic Res. 97:143–156.

[8] Jose PE , Lim BT, Bryant FB. 2012. Does savoring increase happiness? A daily diary study. J Posit Psychol. 7:176–187.

[9] Seidlitz L , Diener E. 1993. Memory for positive versus negative life events: theories for the differences between happy and unhappy persons. J Pers Soc Psychol. 64:654. 8473982 10.1037//0022-3514.64.4.654

[10] de Mello VO , Cheung, F, Inzlicht M. 2022. Twitter use in the everyday life: exploring how Twitter use predicts well-being, polarization, and sense of belonging. 10.1038/s44271-024-00062-z PMC11332209 39242975

[11] Brady WJ , et al 2023. Overperception of moral outrage in online social networks inflates beliefs about intergroup hostility. Nat Hum Behav. 7:917–927. 37037990 10.1038/s41562-023-01582-0

[12] Brady WJ , Crockett MJ, Van Bavel JJ. 2020. The MAD model of moral contagion: the role of motivation, attention, and design in the spread of moralized content online. Perspect Psychol Sci. 15:978–1010. 32511060 10.1177/1745691620917336

[13] Brady WJ , Gantman AP, Van Bavel JJ. 2020. Attentional capture helps explain why moral and emotional content go viral. J Exp Psychol Gen. 149:746–756. 31486666 10.1037/xge0000673

[14] Fan R , Xu KE, Zhao J. 2016. Higher contagion and weaker ties mean anger spreads faster than joy in social media, arXiv, arXiv:1608.03656, preprint: not peer reviewed.

[15] Chang D , Ghim G. 2011. The structure and dynamics of the Korean Twitter network. J Commun Res. 48:59–86.

[16] Goldenberg A , Gross JJ. 2020. Digital emotion contagion. Trends Cogn Sci. 24:316–328. 32160568 10.1016/j.tics.2020.01.009

[17] Milkman KL , Berger J. 2014. The science of sharing and the sharing of science. Proc Natl Acad Sci. 111:13642–13649. 25225360 10.1073/pnas.1317511111 PMC4183177

[18] Gruzd A , Doiron S, Mai P. 2011. Is happiness contagious online? A case of Twitter and the 2010 Winter Olympics. 2011 44^th^ Hawaii International Conference on System Sciences. p. 1–9.

[19] Kraft PW , Krupnikov Y, Milita K, Ryan JB, Soroka S. 2020. Social media and the changing information environment: sentiment differences in read versus recirculated news content. Public Opin Q. 84:195–215.

[20] Bellovary AK , Young NA, Goldenberg A. 2021. Left- and right-leaning news organizations use negative emotional content and elicit user engagement similarly. Affect Sci. 2:391–396. 34423311 10.1007/s42761-021-00046-w PMC8364833

[21] Soroka S , Fournier P, Nir L. 2019. Cross-national evidence of a negativity bias in psychophysiological reactions to news. Proc Natl Acad Sci. 116:18888–18892. 31481621 10.1073/pnas.1908369116 PMC6754543

[22] Soroka S , Carbone M. Gatekeeping, Technology, and Polarization. 2022. In: Oxford Research Encyclopedia of Politics. Oxford: Oxford University Press. https://oxfordre.com/politics/view/10.1093/acrefore/9780190228637.001.0001/acrefore-9780190228637-e-43

[23] Baumeister RF , Bratslavsky E, Finkenauer C, Vohs KD. 2001. Bad is stronger than good. Rev Gen Psychol. 5:323–370.

[24] Zhang H , Qu C. 2020. Emotional, especially negative microblogs are more popular on the web: evidence from an fMRI study. Brain Imaging Behav. 14:1328–1338. 30511115 10.1007/s11682-018-9998-6

[25] Hansen LK , Arvidsson A, Nielsen FÅ, Colleoni E, Etter M. 2011. Good friends, bad news-affect and virality in Twitter. Future Information Technology: 6^th^ International Conference, FutureTech 2011, Loutraki, Greece, June 28-30, 2011, Proceedings, Part II; Springer. p. 34–43.

[26] Berger J , Milkman K. 2010. Social transmission, emotion, and the virality of online content. Whart Res Pap. 106:1–52.

[27] Ruhrmann G , Woelke J, Maier M, Diehlmann N. 2013. Der Wert von Nachrichten im deutschen Fernsehen: Ein Modell zur Validierung von Nachrichtenfaktoren. Schriftenreihe Medienforschung der Landesanstalt für Medien in NRW. VS Verlag für Sozialwissenschaften. Berlin: Springer-Verlag. https://books.google.co.uk/books?id=S6PyBQAAQBAJ.

[28] Peterson S . 1981. International news selection by the elite press: a case study. Public Opin Q. 45:143–163.

[29] Straughan DM . 1989. An experiment on the relation between news values and reader interest. Gaz Leiden Neth. 43:93–107.

[30] Goldenberg A , et al 2020. Beyond emotional similarity: the role of situation-specific motives. J Exp Psychol Gen. 149:138–159. 31192635 10.1037/xge0000625

[31] Wojcik S , Hughes A. 2019. How Twitter users compare to the general public. Pew Res Cent Internet Sci Tech. 156. https://www.pewresearch.org/internet/2019/04/24/sizing-up-twitter-users/

[32] Wojcieszak M , Casas A, Yu X, Nagler J, Tucker JA. 2022. Most users do not follow political elites on Twitter; those who do show overwhelming preferences for ideological congruity. Sci Adv. 8:eabn9418. 10.1126/sciadv.abn9418 PMC9524832 36179029

[33] González-Bailón S , De Domenico M. 2021. Bots are less central than verified accounts during contentious political events. Proc Natl Acad Sci. 118:e2013443118. 10.1073/pnas.2013443118 PMC7980437 33836572

[34] Wang H , Lei K, Xu K. 2015. Profiling the followers of the most influential and verified users on Sina Weibo. 2015 IEEE International Conference on Communications (ICC); IEEE. p. 1158–1163.

[35] Cha M , Haddadi H, Benevenuto F, Gummadi K. 2010. Measuring user influence in Twitter: the million follower fallacy. Proc Int AAAI Conf Web Soc Media. 4:10–17.

[36] Wies S , Bleier A, Edeling A. 2023. Finding goldilocks influencers: how follower count drives social media engagement. J Mark. 87:383–405.

[37] Bossetta M . 2018. The digital architectures of social media: comparing political campaigning on Facebook, Twitter, Instagram, and Snapchat in the 2016 US election. J Mass Commun Q. 95:471–496.

[38] Ross K , Bürger T. 2014. Face to face (book): social media, political campaigning and the unbearable lightness of being there. Polit Sci. 66:46–62.

[39] Furlow NE . 2011. Find us on Facebook: how cause marketing has embraced social media. J Mark Dev Compet. 5:61–64.

[40] Robertson CE , et al 2023. Negativity drives online news consumption. Nat Hum Behav. 7:812–822. 36928780 10.1038/s41562-023-01538-4 PMC10202797

[41] Fine JA , Hunt MF. 2021. Negativity and elite message diffusion on social media. Polit Behav. 1–19. https://link.springer.com/article/10.1007/s11109-021-09740-8

[42] Stieglitz S , Dang-Xuan L. 2013. Emotions and information diffusion in social media—sentiment of microblogs and sharing behavior. J Manag Inf Syst. 29:217–248.

[43] Hutto C , Gilbert E. 2014. VADER: a parsimonious rule-based model for sentiment analysis of social media text. Proceedings of the International AAAI Conference on Web and Social Media. p. 216–225.

[44] Blei DM . 2012. Probabilistic topic models. Commun ACM. 55:77–84.

[45] Nakagawa S , Johnson PC, Schielzeth H. 2017. The coefficient of determination R 2 and intra-class correlation coefficient from generalized linear mixed-effects models revisited and expanded. J R Soc Interface. 14:20170213. 10.1098/rsif.2017.0213 PMC5636267 28904005

[46] Barton K , Barton MK. 2015. Package ‘MuMIn’. Version. 1:439.

[47] Hayes AF . 2017. Introduction to mediation, moderation, and conditional process analysis: a regression-based approach. New York: Guilford Publications.

[48] Gerbner G , Gross L, Morgan M, Signorielli N, Shanahan J. Growing up with television: cultivation processes. 2002. Media effects. UK: Routledge. p. 53–78.

[49] Näsi M , Räsänen P, Hawdon J, Holkeri E, Oksanen A. 2015. Exposure to online hate material and social trust among Finnish youth. Inf Technol People. 28:607–622.

[50] Keipi T , Räsänen P, Oksanen A, Hawdon J, Näsi M. 2018. Exposure to online hate material and subjective well-being: a comparative study of American and Finnish youth. Online Inf Rev. 42:2–15.

[51] Feinberg M , Ford B, Thai S, Gatchpazian A, Lassetter B. 19 September 2020. The political is personal: daily politics as a chronic stressor. PsyArXiv Doi 10, preprint: not peer reviewed.

[52] Frimer JA , et al 2023. Incivility is rising among American politicians on Twitter. Soc Psychol Personal Sci. 14:259–269. 10.1177/19485506221136182 PMC10396794 37545484

[53] Brady WJ , McLoughlin K, Doan TN, Crockett MJ. 2021. How social learning amplifies moral outrage expression in online social networks. Sci Adv. 7:eabe5641. 10.1126/sciadv.abe5641 PMC8363141 34389534

[54] Van Der Helm E , Walker MP. 2012. Sleep and affective brain regulation. Soc Personal Psychol Compass. 6:773–791.

[55] Kelly C , Sharot T. 2023. Knowledge-seeking reflects and shapes well-being.

[56] Ma GWS , Schöne J, Parkinson B. 2022. Social sharing of emotion during the collective crisis of COVID-19. 10.1111/bjop.12729 39215960

[57] Stuart EA , King G, Imai K, Ho D. 2011. MatchIt: nonparametric preprocessing for parametric causal inference. J Stat Softw. 42:1–28.

[58] Ribeiro FN , Araújo M, Gonçalves P, André Gonçalves M, Benevenuto F. 2016. SentiBench—a benchmark comparison of state-of-the-practice sentiment analysis methods. EPJ Data Sci. 5:1–29.

[59] Thelwall M , Buckley K, Paltoglou G, Cai D, Kappas A. 2010. Sentiment strength detection in short informal text. J Am Soc Inf Sci Technol. 61:2544–2558.

[60] Berger J , Packard G. 2018. Are atypical things more popular? Psychol Sci. 29:1178–1184. 29671695 10.1177/0956797618759465

[61] Blei DM , Lafferty J. 2009. Topic models. Text mining: theory and applications. Boca Raton, FL: CRC Press.

[62] Grün B , Hornik K, Grün MB. 2017. Package ‘topicmodels’. Retrieved January 1, 2018.

[63] Jacobi C , Van Atteveldt W, Welbers K. 2016. Quantitative analysis of large amounts of journalistic texts using topic modelling. Digit J. 4:89–106.

[64] Chang J , Gerrish S, Wang C, Boyd-Graber J, Blei D. 2009. Reading tea leaves: how humans interpret topic models. Adv Neural Inf Process Syst. 22:288–296. https://proceedings.neurips.cc/paper_files/paper/2009/file/f92586a25bb3145facd64ab20fd554ff-Paper.pdf.

[65] Pew Research Centre . “The political content in users’ tweets and the accounts they follow”.

[66] Barberá P . 2015. Birds of the same feather tweet together: Bayesian ideal point estimation using Twitter data. Polit Anal. 23:76–91.

[67] Z. Wang , et al 2019. Demographic inference and representative population estimates from multilingual social media data. The World Wide Web Conference. p. 2056–2067.

