# Supplementary material for: Negative expressions are shared more on Twitter for public figures than for ordinary users
Source: PNAS Nexus. 2023 Jul 6;2(7):pgad219. doi: 10.1093/pnasnexus/pgad219 (PMC10338895; doi:10.1093/pnasnexus/pgad219)
Supplement: pgad219_Supplementary_Data [file pgad219_supplementary_data.docx]

**Negative Expressions are Shared more on Twitter for Public Figures than for Ordinary Users**

**(Supplementary Information)**

[1. Analysis Using the Full Sample 1](#_Toc133518041)

[Association between Sentiment and Retweets for Public Figures versus Ordinary Users. 2](#_Toc133518042)

[Differences between Public Figures and Ordinary Users. 3](#_Toc133518043)

[Association between User Characteristics and Negativity Sharing. 4](#_Toc133518044)

[Number of Followers 4](#_Toc133518045)

[Proportion of Political Tweets 6](#_Toc133518046)

[Parallel Mediation Analysis. 7](#_Toc133518047)

[2. Sentiment Tool Comparison 8](#_Toc133518048)

[3. Topics Modelling Detailed Information 10](#_Toc133518049)

[Political Topic Presented in the Manuscript. 10](#_Toc133518050)

[Political Topic Assessed Using Topics Modelling k = 50 and PCA 12](#_Toc133518051)

[Creating Alternative Classification 12](#_Toc133518052)

[Comparing Alternative Classification to Model presented in the Manuscript 13](#_Toc133518053)

[Repeating Analysis Using Alternative Classification 14](#_Toc133518054)

[4. Influence of Number of Followers for Ordinary Users 16](#_Toc133518055)

[5. Analysing Different Types of Verified Users 17](#_Toc133518056)

[6. Average Emotion Expressed by Users 21](#_Toc133518057)

[7. General Additive Model of Sentiment and Retweets for Public Figures and Ordinary Users 24](#_Toc133518058)

[GAM for Public Figures. 24](#_Toc133518059)

[GAM for Ordinary Users 25](#_Toc133518060)

[8. References 27](#_Toc133518061)

# Analysis Using the Full Sample

The goal of this analysis was to replicate the findings reported in the main manuscript using the 2,674,418 tweets of all users, including 2,246,068 tweets produced by 39,241 public figures and 428,350 tweets from 6,677 ordinary users. We repeated every analysis as reported in the main manuscript using this full sample, except for the *Association between User Characteristics and Negativity Sharing* section*.* Differences in analysis were described in the corresponding sections.

## Association between Sentiment and Retweets for Public Figures versus Ordinary Users.

For this analysis, the dependent variable was content sharing, measured by the number of retweets (log+1-transformed). For the independent variable, we used the continuous sentiment score from VADER (-1 extremely negative to +1 extremely positive). We fitted a quadratic mixed model to predict the number of retweets using a quadratic function of the continuous sentiment score between -1 and 1.

**Table S1.** Quadratic mixed model with five factors (Sentiment^2^, Sentiment^1^, User_type, Sentiment^1^ * User_type, Sentiment^2^ * User_type) and number of retweets (log+1) as the dependent variable.

| **Fixed Effects** | | | | | |
| --- | --- | --- | --- | --- | --- |
|  | Estimate | *SE* | 95% CI | *t* | *p* |
| Intercept | 0.20 | 0.11 | 0.18 – 0.22 | 18.63 | .000 |
| Sentiment^1^ | 0.0048 | 0.002 | -0.00095 – 0.010 | 1.63 | .10 |
| Sentiment^2^ | 0.048 | 0.0055 | 0.037 – 0.059 | 8.78 | .000 |
| User_type:Public Figure | 0.61 | 0.011 | 0.59 – 0.63 | 51.55 | .000 |
| Sentiment^1^ × User_type:Public Figure | -0.10 | 0.0032 | -0.10 – -0.095 | -31.23 | .000 |
| Sentiment^2^ × User_type:Public Figure | 0.15 | 0.0060 | 0.14 – 0.16 | 25.90 | .000 |
| **Random Effects** | | | | | |
|  | |  | Variance | *SD* |  |
| User_id (Intercept) | |  | 0.82 | 0.90 |  |
| Residual | |  | 0.64 | 0.80 |  |
| **Model Fit** | | | | | |
| *R*^2^ | |  | Marginal | Conditional | |
|  | |  | 0.037 | 0.57 | |
| Model equation: log(retweets+1) ~ poly(compound,2, raw = TRUE) * user_type + (1 \| User) | | | | | |

*Notes.* Model fit was calculated using the R package MuMIn (Barton & Barton, 2015) based on the paper of Nakagawa, Johnson, and Schielzeth (2017).


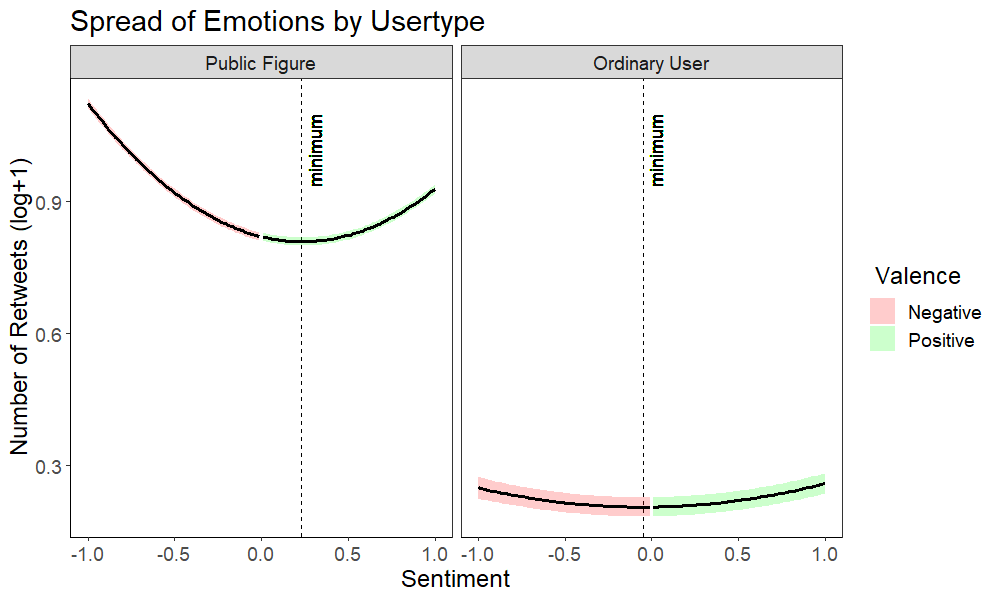


**Figure S1.** Number of retweets as a function of sentiment and user type. The results suggest that stronger sentiment is associated with more retweets for both types of users. The local minimum for public figures is reached with a more positive emotional tweet, indicating that negativity is more strongly positively associated with number of retweets for public figures than for ordinary users. Public figures also receive more retweets for neutral content than ordinary users.

## Differences between Public Figures and Ordinary Users.

We tested if public figures had more followers, and a higher proportion of political tweets than ordinary users, using the aggregated user characteristics measures. These were the log+1 transformed number of followers of a user, and the proportion of political tweets defined as the number of tweets identified as political tweets by the topics modelling divided by the number of total tweets. We used a simple linear regression predicting the two user characteristics with a dummy coded variable for verification status.

As reported in the main manuscript, the analysis using the full dataset replicated the finding in the main manuscript that public figures had more followers (*b* = 4.33 [4.28, 4.37], SE = 0.022, *t* (45911) = 190.2, *p* < .001, *R*^2^ = .44, see Figure 2S.A), and produced content that contained approximately 2% more political content (*b* = 0.019 [0.015, 0.025], SE = 0.0020, *t* (45911) = 9.80, *p* < .001, *R*^2^ = .0020, see Figure 2S.B) than ordinary users.


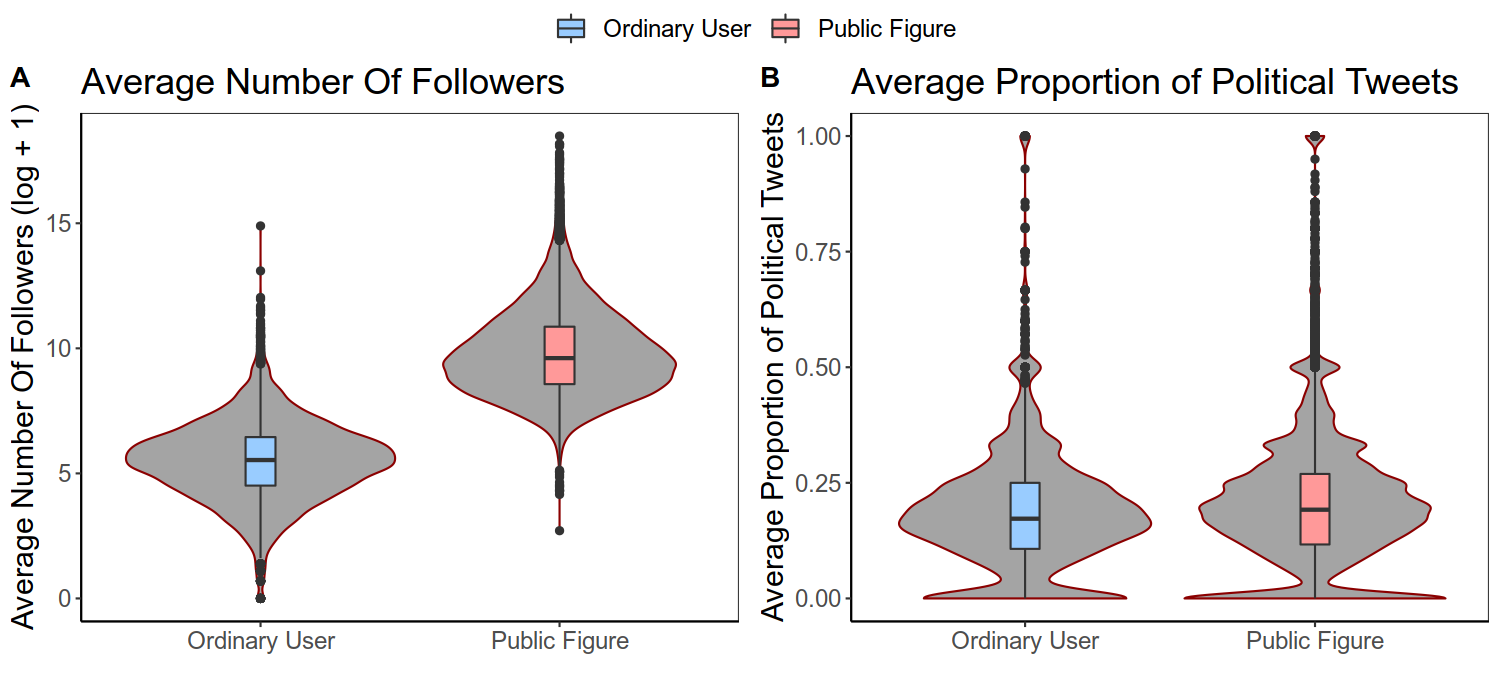


**Figure S2.** Differences between public figures and ordinary users. Results suggest that public figures have more followers than ordinary users (Panel A), and produce a higher proportion of political tweets (Panel B).

## Association between User Characteristics and Negativity Sharing.

We tested if user characteristics interacted with the relationship between sentiment and the number of retweets. We again used a quadratic mixed model to predict the number of retweets using a quadratic function of the continuous sentiment score. We included each user characteristic as an interaction factor with sentiment in two models separately and added a random intercept for users to account for baseline differences in the average number of retweets that they get. In contrast to the analysis reported in the main manuscript, we added a covariate for the different user types into the models to account for the differences in sample size, as the current sample contains more verified users than ordinary users. This should avoid that the effect of user characteristics is driven by the more frequent verified users.

### Number of Followers

**Table S2.** Quadratic mixed model with six factors (Sentiment^2^, Sentiment^1^, Number_of_Followers, User_type, Sentiment^1^ * Number_of_Followers, Sentiment^2^ * Number_of_Followers) and number of retweets (log+1) as the dependent variable.

| **Fixed Effects** | | | | | |
| --- | --- | --- | --- | --- | --- |
|  | Estimate | *SE* | 95% CI | *t* | *p* |
| Intercept | 1.31 | 0.012 | 1.28 – 1.33 | 101.70 | .000 |
| Sentiment^1^ | -0.081 | 0.0012 | -0.083 – -0.078 | -65.10 | .000 |
| Sentiment^2^ | 0.17 | 0.0022 | 0.17 – 0.18 | 79.39 | .000 |
| Number_of_Followers | 0.66 | 0.0051 | 0.65 – 0.67 | 129.38 | .000 |
| User_type:Public Figure | -0.45 | 0.013 | -0.47 – -0.42 | -33.98 | .000 |
| Sentiment^1^ × Number_of_Followers | -0.038 | 0.0012 | -0.041 – -0.036 | -31.97 | .000 |
| Sentiment^2^ × Number_of_Followers | 0.047 | 0.0022 | 0.043 – 0.052 | 21.41 | .000 |
| **Random Effects** | | | | | |
|  | |  | Variance | *SD* |  |
| User_id (Intercept) | |  | 0.58 | 0.76 |  |
| Residual | |  | 0.64 | 0.80 |  |
| **Model Fit** | | | | | |
| *R*^2^ | |  | Marginal | Conditional | |
|  | |  | 0.21 | 0.58 | |
| Model equation: log(retweets+1) ~ poly(compound,2, raw = TRUE) * scaled(Number_of_Followers) + (1 \| User) | | | | | |

*Notes.* Model fit was calculated using the R package MuMIn (Barton & Barton, 2015) based on the paper of Nakagawa et al. (2017).


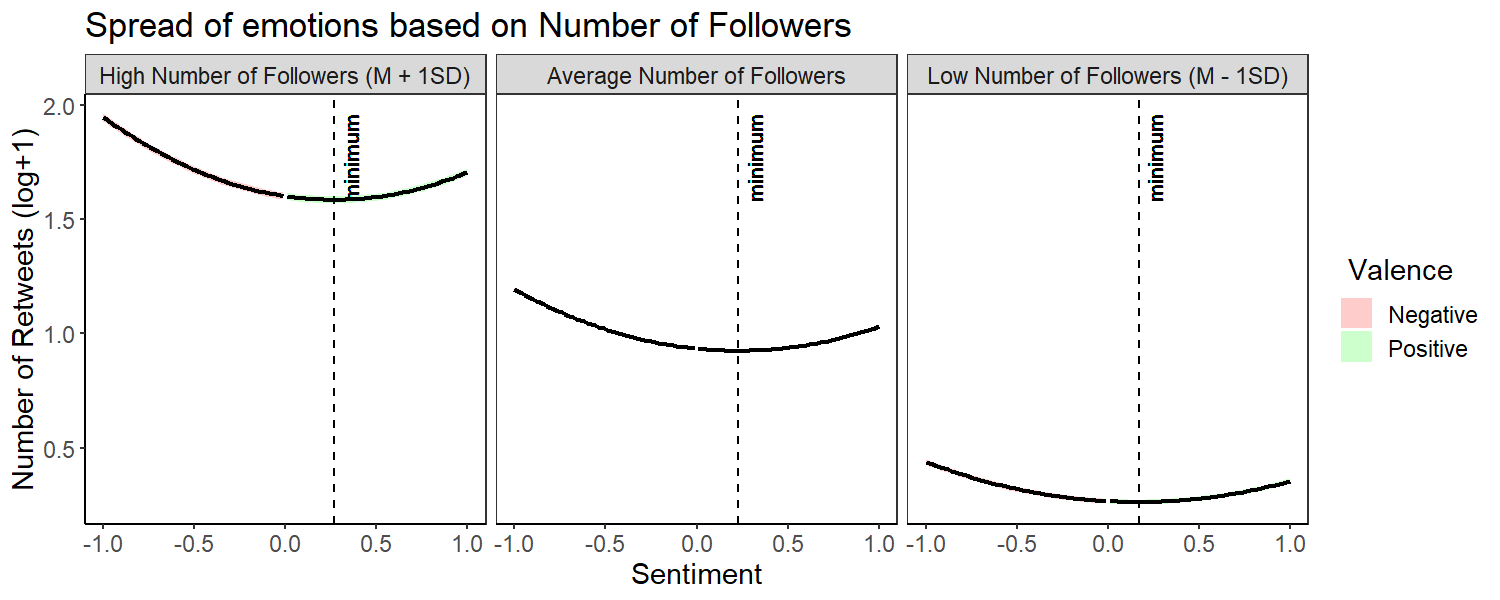


**Figure S3.** *The number of retweets as a function of sentiment and number of followers*. To visualize the interaction of two continuous variables (sentiment and the number of followers), the panels show the mean number of followers in the middle panel as well as the mean plus and minus one standard deviation (left and right panel respectively). The results showed that an increase in the number of followers strengthens the link between sentiment and content sharing. Negativity sharing was stronger for the content of users with more followers.

### Proportion of Political Tweets

**Table S3.** Quadratic mixed model with six factors (Sentiment^2^, Sentiment^1^, Political_Tweets, User_type, Sentiment^1^ * Political_Tweets, Sentiment^2^ * Political_Tweets) and number of retweets (log+1) as the dependent variable.

| **Fixed Effects** | | | | | |
| --- | --- | --- | --- | --- | --- |
|  | Estimate | *SE* | 95% CI | *t* | *p* |
| Intercept | 0.19 | 0.011 | 0.17 – 0.21 | 17.55 | .000 |
| Sentiment^1^ | -0.077 | 0.0012 | -0.080 – -0.075 | -61.93 | .000 |
| Sentiment^2^ | 0.17 | 0.0022 | 0.17 – 0.18 | 77.31 | .000 |
| Political_Tweets | 0.021 | 0.0027 | 0.015 – 0.026 | 7.73 | .000 |
| User_type:Public Figure | 0.63 | 0.011 | 0.60 – 0.65 | 53.17 | .000 |
| Sentiment^1^ × Political_Tweets | -0.013 | 0.0013 | -0.015 – -0.010 | -10.08 | .000 |
| Sentiment^2^ × Political_Tweets | 0.0059 | 0.0023 | 0.0013 – 0.010 | 2.54 | .000 |
| **Random Effects** | | | | | |
|  | |  | Variance | *SD* |  |
| User_id (Intercept) | |  | 0.82 | 0.90 |  |
| Residual | |  | 0.64 | 0.80 |  |
| **Model Fit** | | | | | |
| *R*^2^ | |  | Marginal | Conditional | |
|  | |  | 0.037 | 0.58 | |
| Model equation: log(retweets+1) ~ poly(compound,2, raw = TRUE) * scaled(Political_Tweets) + (1 \| User) | | | | | |

*Notes.* Model fit was calculated using the R package MuMIn (Barton & Barton, 2015) based on the paper of Nakagawa et al. (2017).


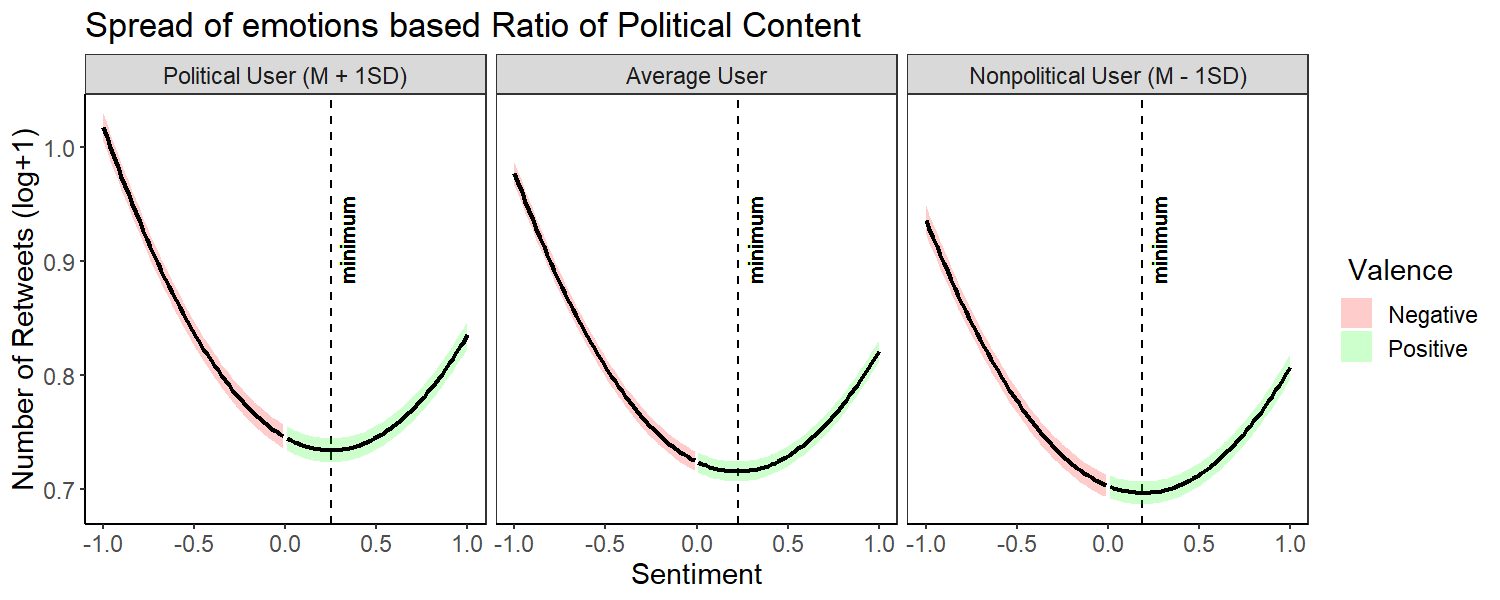


**Figure S4.** *The number of retweets as a function of sentiment* *and proportion of political tweets*. To visualize the interaction of two continuous variables (sentiment and proportion of political tweets), the panels show the predicted association between sentiment and the number of retweets at the mean proportion of political tweets in the middle panel as well as the mean plus and minus one standard deviation (left and right panel respectively). The results indicate that tweets produced by users with a higher proportion of political tweets show stronger associations between sentiment and content sharing and between negativity and content sharing.

## Parallel Mediation Analysis.

Finally, we repeated the parallel mediation analysis assessing two potential mediators of the effects of user type (public figures vs. ordinary users) on sharing of their negative content (Hayes, 2017) as described in the manuscript. The potential mediators were the two user characteristics, i.e., number of followers, and the proportion of political tweets. We again calculated the dependent variable that quantified the extent to which an increase in negativity was associated with more retweets for every individual user in our dataset (see manuscript for all details). We used the PROCESS v4 macro for RStudio by Hayes (2017) to conduct the parallel mediation analysis.

As reported above, user type correlated positively with number of followers (a_1_ = 4.32 [4.28, 4.37], SE = 0.022, *t* (45913) = 190.032, *p* <.001, *R*^2^ = 0.09, see Figure 5S), and the proportion of political tweets (a_2_ = 0.020 [0.016, 0.024], SE = 0.0020, *t* (45913) = 6.56, *p* < .001, *R*^2^ = 0.09). A 95% bias-corrected confidence interval based on 10,000 bootstrap samples indicated that the sampled indirect effects through number of followers (a_1_b_1_ = 0.10, SE = 0.0024), holding all other mediators constant, were consistently above zero (0.098 - 0.10). The indirect effects through proportion of political tweets were consistently above zero (a_3_b_3_  = 0.0006 [0.0001 - 0.0008], SE = 0.0010). The direct total affect in the absence of the mediator yielded a significant association between verification status and negativity sharing (c = 0.075 [0.071, 0.079], SE = 0.0021, *t* (45913) = 36.01, *p* <.001, R^2^ = 0.09). In contrast to the findings in the main manuscript, the total effect of user type on negativity sharing was also significant after controlling for the mediators (c’ = -0.028 [-0.034 - -0.023], SE = 0.0027, *t* (45913) = -10.67, , *p* <.001, R^2^ = 0.09). These results from the parallel mediation analysis suggest that user type only had an effect on negativity sharing, but was partially mediated by the number of followers and proportion of political tweets.


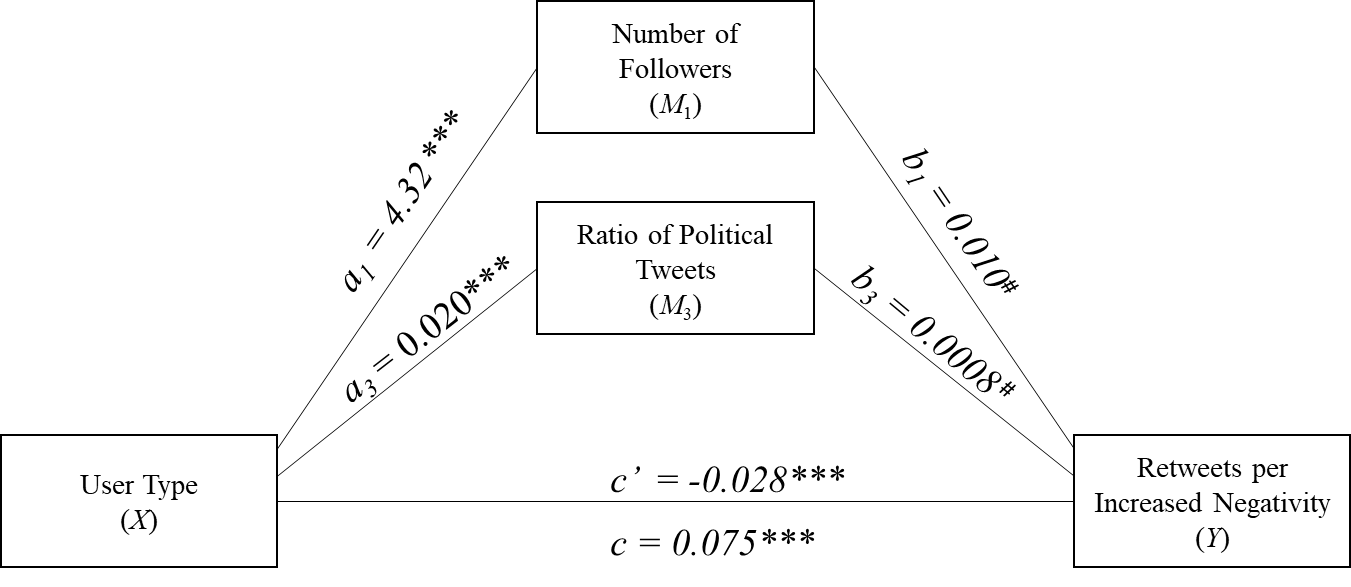


**Figure S5.** *Parallel mediation analyzes the effect of user type on negativity sharing via three different user characteristics*. User type positively predicts each of the user characteristics: number of followers, and proportion of political tweets. The indirect effects of user type on negativity sharing via the number of followers and proportion of political tweets were both significant.

# Sentiment Tool Comparison

To test whether VADER is the best sentiment analysis tool for the dataset, we first tested the correlation between VADER and another commonly used sentiment analysis tool, SentiStrength (Thelwall, Buckley, & Paltoglou, 2012). SentiStrength is comprised of two independent sentiment scores, one for positive language and the other for negative language. We computed a total sentiment score by subtracting a tweets negative sentiment score from the positive sentiment score. The correlation of the total sentiment scores of VADER and SentiStrength was r = 0.56 (95%CI = 0.55 - 0.57, *t*(855713)= 633.99, *p* < .001).

We repeated the analysis testing whether negativity is more positively associated with shares for public figures than for ordinary users by using the identical model as for the VADER analysis. Namely, we predicted the log-modulus transformed number of retweets by the SentiStrength sentiment score and its interaction with user type using a quadratic mixed model with a random intercept of individual users.

**Table S4.** Quadratic mixed model with five factors (SentiStrength_Sentiment^2^, SentiStrength_Sentiment^1^, User_type, SentiStrength_Sentiment^1^ * User_type, SentiStrength_Sentiment^2^ * User_type) and number of retweets (log+1) as the dependent variable.

| **Fixed Effects** | | | | | |
| --- | --- | --- | --- | --- | --- |
|  | Estimate | *SE* | 95% CI | *t* | *p* |
| Intercept | 0.20 | 0.0090 | 0.18 – 0.22 | 22.4 | .000 |
| SentiStrength_Sentiment^1^ | -0.0035 | 0.00080 | -0.0051 – -0.0019 | -4.43 | .000 |
| SentiStrength_Sentiment^2^ | -0.00015 | 0.00041 | -0.00097 – 0.00065 | -0.37 | .71 |
| User_type:Public Figure | 0.61 | 0.012 | 0.58 – 0.63 | 49.53 | .000 |
| SentiStrength_Sentiment^1^ × User_type:Public Figure | -0.036 | 0.0011 | -0.039 – -0.034 | -31.36 | .000 |
| SentiStrength_Sentiment^2^ × User_type:Public Figure | 0.0025 | 0.00059 | 0.0014 – 0.0037 | 4.34 | .000 |
| **Random Effects** | | | | | |
|  | |  | Variance | *SD* |  |
| User_id (Intercept) | |  | 0.53 | 0.73 |  |
| Residual | |  | 0.46 | 0.68 |  |
| **Model Fit** | | | | | |
| *R*^2^ | |  | Marginal | Conditional | |
|  | |  | 0.087 | 0.57 | |
| Model equation: log(retweets+1) ~ poly(SentiStrength, raw = TRUE) * user_type + (1 \| User) | | | | | |

*Notes.* Model fit was calculated using the R package MuMIn (Barton & Barton, 2015) based on the paper of Nakagawa et al. (2017).


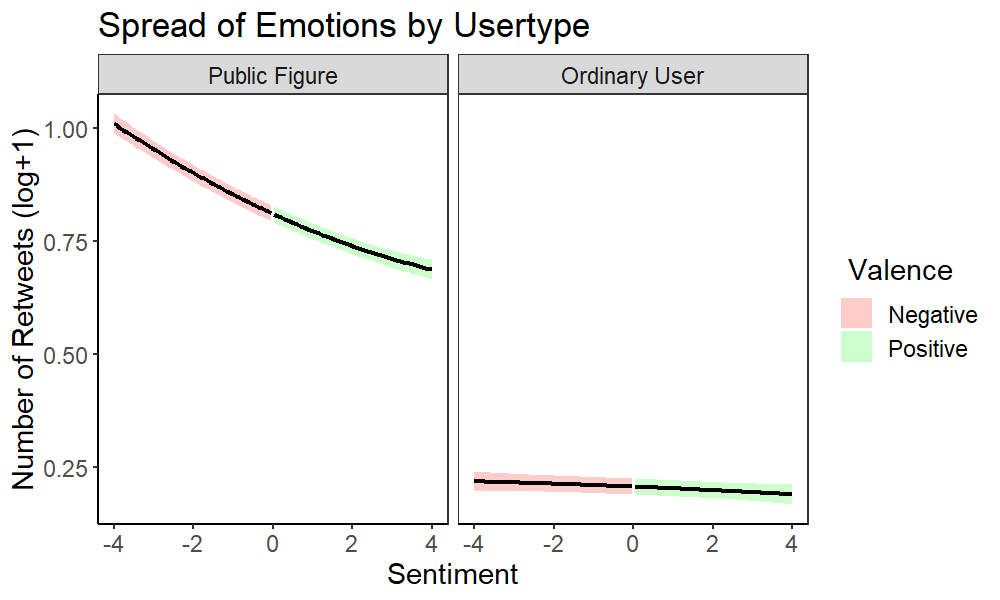


**Figure S6.** *The number of retweets as a function of sentiment as measured by SentiStrength*. The results showed, more negativity is associated with an increase in the number of retweets. This effect was stronger for public figures than for ordinary users, replicating the main finding of the manuscript.

Finally, we compared the model fit between the models using VADER compared to SentiStrength. The model using VADER had the overall better model fit (AIC = 1,818,927) in comparison to SentiStrength (AIC = 1,819,941), we therefore decided to use VADER for all other analysis.

# Topics Modelling Detailed Information

## Political Topic Presented in the Manuscript.

This section is meant to provide more details regarding the procedure applied to determine the political topic used to classify political tweets. We analysed the meaning of each of the 5 topics to find the one that is about politics. In order to understand the meaning of a topic, we first analysed the overarching theme of the words that have the highest probability to be generated by a certain topic, which is expressed by the β-score. After manually inspecting the 5 topics, we identified one topic that seemed to generate politics related word with a higher likelihood (Table 5S). A full list of the most common words per topics is available on the OSF repository (topic_modell_words_5t.csv). We confirmed the quality of the topic classification by inspecting the tweets that had the highest likelihood of containing this topic as indicated by the γ-score (Table 6S).

**Table S5.** *The 20 most frequent unigrams for the political topic.* This table shows the words showing the biggest $\beta$, which indicates the frequency of a word in a given topic. We concluded that this topic based on the words most frequent in this topic is related to politics

| Political Topic | β-score |
| --- | --- |
| Trump | 0.0079 |
| time | 0.0056 |
| day | 0.0052 |
| feel | 0.0047 |
| talk | 0.0045 |
| vote | 0.0044 |
| week | 0.0041 |
| happen | 0.0040 |
| share | 0.0038 |
| win | 0.0038 |
| life | 0.0037 |
| shut-down | 0.0037 |
| people | 0.0036 |
| live | 0.0036 |
| president | 0.0035 |
| update | 0.0032 |
| city | 0.0031 |
| learn | 0.0030 |
| shutdown | 0.0030 |

**Table S6. Most political tweets:** Tweets with the highest γ-score for the above mentioned political topic, meaning that these tweets should contain political content with the highest likelihood according to our topic modelling analysis.

| No. | Tweet content | γ-score |
| --- | --- | --- |
| 1. | McGurk decry 'total reversal' of # us policy on # syria | 0.56 |
| 2. | Sexual harassment and pay disparity in his campaign complicate Bernie Sander. The former presidential candidate's desire to return to the ring is complicated by several testimonies revealed by 'The New York Times' | 0.56 |
| 3. | the man accuse in the fatal shoot of lsu basketball player Wayde Sims have be indict on a murder charge | 0.47 |
| 4. | API investor Andy Gracey say a proposal from the drug wholesaler to merge with sigma healthcare throw a lifeline to a under - pressure rival, @ jessigardner report. | 0.44 |
| 5. | hook, line, and sinker - what do you know about # phishing? it's a effective and often successful practice, so how do you prevent it? nleefvejc # cybersecurity | 0.43 |
| 6. | turkey: pres. erdogan blast u. s. change in syria withdrawal plan | 0.25 |
| 7. | Opinion: the shutdown, which change the ceremony, have also deeply affect a group # mlk advocate for - people experience poverty. | 0.24 |
| 8. | The partial government shutdown, now the under president donald trump, be the long shutdown in u. s. history as of today ( jan. ). its impact be far reach in # washington, strain earthquake research and, soon, food assistance. | 0.24 |
| 9. | There's plenty of speculation over the reason u. s. president Donald Trump have call a prime - time national address, many believe it's a new attempt to end the stalemate over his mexican border wall. | 0.24 |
| 10. | Probe governor Obado over new murder scheme | 0.24 |

## Political Topic Assessed Using Topics Modelling k = 50 and PCA

### Creating Alternative Classification

The number of topics in topics modelling is selected manually. As mentioned in the manuscript, we tried to identify the minimum number of topics that contained mostly political words in order to avoid having multiple politics topics. As an alternative test, instead of using the smallest number of topics that includes a political topic, we used larger numbers of topics (k = 50), potentially including multiple but more specific political topics. The manual assessment of the topic meanings confirmed that there were multiple topics related to politics (e.g. see Table S7 for the three topics which are associated with the final politics variable as determined by the following procedure, see topic_modell_words_50t.csv for full list of words in topics). However, in order to conduct the analysis using a single politics classification variable, we used Principle Component Analysis (PCA) to reduce the dimensionality of the 50 topics by clustering those share the most variance with the goal to identify one component that is comprised of political topics. We decided to conduct a PCA analysis with 5 components to match the number of components in the topic modelling presented in the manuscript. To identify the component that represents political content, we analysed the semantic meaning of the topics that had the highest factor loadings for each component. The political component was therefore the one that had the highest factor loadings from various political topics (Table S8). We then classified a tweet as political if the component containing the political topics had the factor score compared to the other four components.

**Table S7.** *The 5 most frequent unigrams for the political topics that contribute most to the political component.* This table shows the words showing the biggest $\beta$, which indicates the frequency of a word in a given topic. We concluded that this topic based on the words most frequent in this topic is related to politics

| Topic | Most Frequent Word | β-score |
| --- | --- | --- |
| A | Trump | 0.061 |
| A | wall | 0.052 |
| A | president | 0.039 |
| A | border | 0.032 |
| A | news | 0.027 |
| B | shutdown | 0.018 |
| B | government | 0.010 |
| B | question | 0.0089 |
| B | Trump | 0.0081 |
| B | fund | 0.0080 |
| C | government | 0.023 |
| C | people | 0.018 |
| C | time | 0.015 |
| C | color | 0.013 |
| C | risk | 0.012 |

**Table S8.** *Factor contribution of the political topics to the political factor***:** For each political topic described in the previous table, this table shows how much they contribute to the political component that is used for classification (in %).

| Political Component | Topic Contribution |
| --- | --- |
| Topic A | 16.94 |
| Topic B | 8.74 |
| Topic C | 7.65 |

### Comparing Alternative Classification to Model presented in the Manuscript

We tested how correlated the classification of political tweets was using the method described in the manuscript (topic modelling, k = 5) with the above-mention method (topic modelling, k = 50, PCA =5). The correlation between the estimates was r = 0.14 (95%CI = 0.55 - 0.57, *t*(857740)= 138.7, *p* < .001). We also tested how correlated the estimation of the user characteristics, proportion of political tweets (calculated as the number of political tweets compared to all tweets), was using both tools correlated with r = 0.22 (95%CI = 0.21 - 0.23, *t*(45911)= 48.28, *p* < .001).

### Repeating Analysis Using Alternative Classification

We repeated the analysis regarding the proportion of political tweets. First, we compared the difference in the proportion of political tweets of public figures compared to ordinary users. Before also testing how the proportion of political tweets influences the likelihood of negativity being shared for someone.

In line with the findings presented in the main manuscript, public figures produced more political content (*b* = 0.04 [0.037, 0.046], SE = 0.0022, *t* (45911) = 18.86, *p* < .001, *R*^2^ = .0076, see Figure S7) than ordinary users.


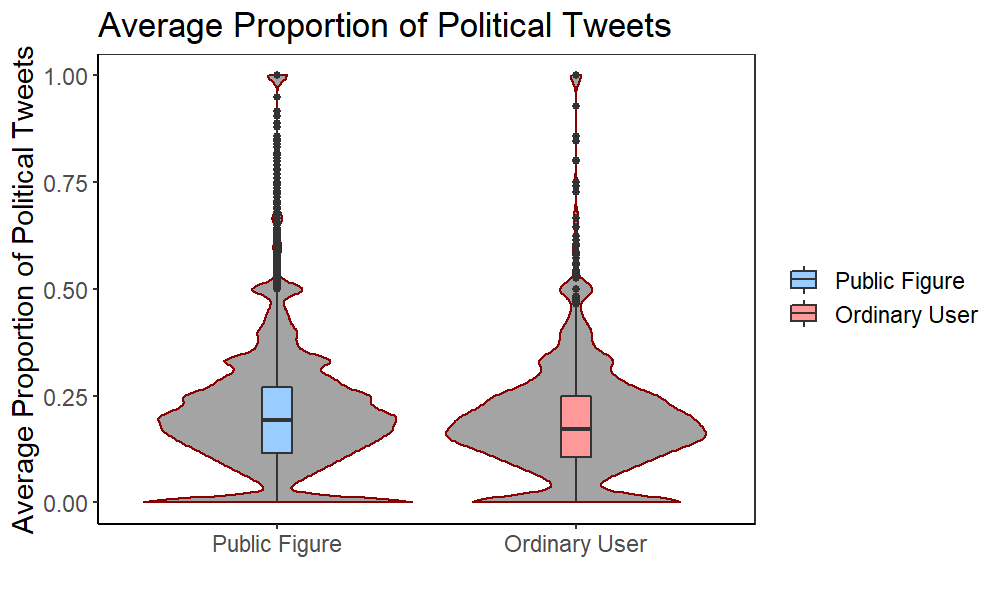


**Figure S7.** *Differences between public figures and ordinary users*. Results suggest that public figures produce around 4% more political content than ordinary users.

We again used a quadratic mixed model to predict the number of retweets using a quadratic function of the continuous sentiment score. We included the alternative estimate for proportion of political content (topic model, k = 50, PCA = 5) as an interaction factor with sentiment in the model and added a random intercept for users to account for baseline differences in the average number of retweets that they get.

**Table S9.** Quadratic mixed model with five factors (Sentiment^2^, Sentiment^1^, Political_Tweets, Sentiment^1^ * Political_Tweets, Sentiment^2^ * Political_Tweets) and number of retweets (log+1) as the dependent variable.

| **Fixed Effects** | | | | | |
| --- | --- | --- | --- | --- | --- |
|  | Estimate | *SE* | 95% CI | *t* | *p* |
| Intercept | 0.50 | 0.0073 | 0.48 – 0.51 | 68.79 | .000 |
| Sentiment^1^ | -0.048 | 0.0018 | -0.051 – -0.044 | -25.94 | .000 |
| Sentiment^2^ | 0.13 | 0.0034 | 0.12 – 0.13 | 38.16 | .000 |
| Political_Tweets | 0.093 | 0.0056 | 0.082 – 0.10 | 16.73 | .000 |
| Sentiment^1^ × Political_Tweets | -0.020 | 0.0017 | -0.023 – -0.016 | -11.75 | .000 |
| Sentiment^2^ × Political_Tweets | 0.011 | 0.0032 | 0.0044 – 0.017 | 3.38 | .000 |
| **Random Effects** | | | | | |
|  | |  | Variance | *SD* |  |
| User_id (Intercept) | |  | 0.82 | 0.90 |  |
| Residual | |  | 0.64 | 0.80 |  |
| **Model Fit** | | | | | |
| *R*^2^ | |  | Marginal | Conditional | |
|  | |  | 0.0097 | 0.58 | |
| Model equation: log(retweets+1) ~ poly(compound,2, raw = TRUE) * scaled(Political_Tweets) + (1 \| User) | | | | | |

*Notes.* Model fit was calculated using the R package MuMIn (Barton & Barton, 2015) based on the paper of Nakagawa et al. (2017).


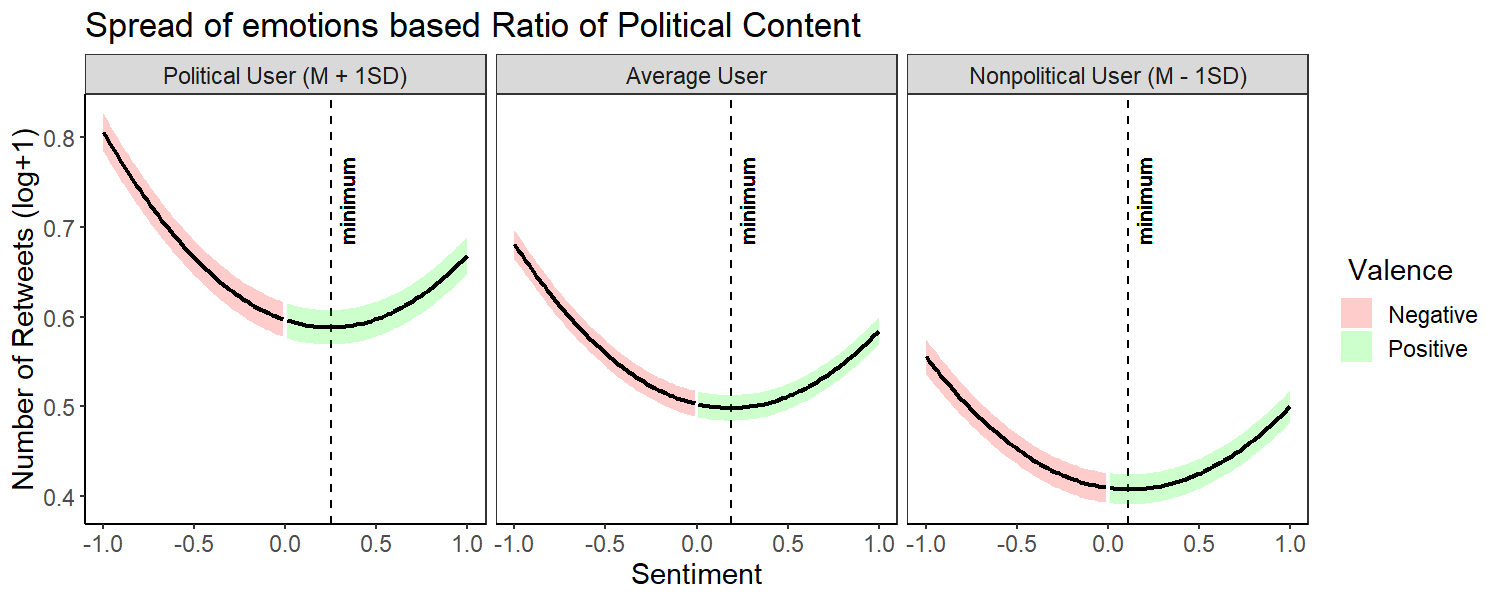


**Figure S8.** *The number of retweets as a function of sentiment and proportion of political tweets*. To visualize the interaction of two continuous variables (sentiment and proportion of political tweets), the panels show the predicted association between sentiment and the number of retweets at the mean proportion of political tweets in the middle panel as well as the mean plus and minus one standard deviation (left and right panel respectively). The results indicate that tweets produced by users with a higher proportion of political tweets show stronger associations between sentiment and content sharing and between negativity and content sharing.

# Influence of Number of Followers for Ordinary Users

As described in the section titled "**Differences between Public Figures and Ordinary Users**", verified users tend to have a substantially larger number of followers than ordinary users, to the extent that this difference may have influenced the observed effect of follower count on negativity sharing. To address this concern, we attempted to replicate the findings by matching the two user types based on their number of followers. However, this is challenging as verified users typically have a much larger following than ordinary users. As a result, the sample size after matching is extremely small and unrepresentative of their respective groups. To illustrate this point, we determined the percentage of overlap between the two groups, by first estimating the density functions for each group using the KDE method in R with the '*bw*' parameter controlled by the *nrd* method (Duong, 2007). After obtaining the density estimates for both groups, we then calculated the area of overlap between them using the trapezoidal rule. We found that the two groups had a percentage overlap of 16.8%. Given our sample size of 6,678 ordinary users, this would result in a subsample of only 1122 users, who are not representative of either ordinary users or their matched verified counterparts. We found that the two groups had a percentage overlap of 16.8%. Given our sample size of 6,678 ordinary users, this would result in a subsample of only 1,122 users, who are not representative of either ordinary users or their matched verified counterparts.

Despite these considerations, we matched ordinary users and public figures along two dimensions – degree of activity and amount of followers – using propensity score matching. We selected the top 10% percentile of ordinary users in terms of followers (660) and matched them with verified users with the closest number of followers and number of total tweets. This number is smaller than the 16.8% reported above as we matched users also based on activity as well. A t-test showed that the number of followers between the two subsamples did not differ (t(829.31) = -0.37, p = .70). We then repeated the exact analysis described in the section “**Association between Sentiment and Retweets for Public Figures versus Ordinary Users**” using a matched sample. Meaning, we predicted the number or number of retweets (log+1-transformed) by the interaction between the continuous sentiment score from VADER (-1 extremely negative to +1 extremely positive) and user type using a quadratic mixed model.

Results of the interactions between user type and sentiment suggested that following matching, that for ordinary users negativity was more strongly associated with sharing than positivity (*a* = -0.019 [-0.029, -0.0098], *SE* = 0.0049, t (183057.85) = -3.96, *p* < .001, *R*^2^ = .006). This effect of negativity on retweets of ordinary users was still smaller than for public figures (*a* = -0.015 [-0.028, -0.0023], *SE* = 0.0067, t (183096.86) = -2.30, *p* = .023, *R*^2^ = .006). Similarly, emotional intensity was also not as strongly associated with an increase in number of retweets for ordinary users than for public figures (*b* = -0.0045 [-0.028, 0.019], *SE* = 0.012, t (183022.58) = -0.36, *p* < .001, *R*^2^ = .006).

As this sample of ordinary users is unrepresentative for its user type, we wanted to confirm that the effect of number of followers can be found for all ordinary users not just the ones with large number of followers. Thus, we repeated the analysis that tests the influence of number of followers on the subsample of all ordinary users. Specifically, we used a quadratic mixed model to predict the number of retweets based on the continuous sentiment score for the subsample of all ordinary users. We included the number of followers as an interaction factor with sentiment in the model, and added a random intercept for users to account for baseline differences in their average number of retweets. By including all ordinary users in this analysis, we could still examine the influence of number of followers on the sharing of negativity while avoiding the issues of small and unrepresentative subsamples.

We found that by only looking at this subsample, there was a stronger association between sentiment and number of retweets (*a* = 0.058 [0.049, 0.066], *SE* = 0.0042, t (424206.45) = 13.87, *p* < .001, *R*^2^ = .017), and between negativity and number of retweets (*b* = -0.020 [-0.024, -0.016], *SE* = 0.0022, t (424342.72) = -9.07, *p* < .001, *R*^2^ = .017) for tweets produced by ordinary users with more followers. This finding supports our main argument that the reason for the increased tendency of users' negativity to be shared is due to the number of followers, regardless of user type.

These findings provide further insight into the influence of the number of followers on the sharing of negativity by clarifying two points. On the one hand increase in followers does lead to a stronger association between negativity and sharing for all user types. On the other hand, this association is stronger for public figures, even when matched to ordinary users by follower numbers. We hope that this helps a bit in further interpreting the results.

# Analysing Different Types of Verified Users

On Twitter, various types of public figures can obtain verification status, including politicians, journalists, celebrities to athletes. Some of verified users are individuals while others represent organizations such as companies, universities or government institutions. This raises the question whether the main finding, namely, that negative content is shared more for verified users, hold true for all types of verified users. Nonetheless, we hypothesize that the tendency for negative content to be shared more among verified users is due to their common characteristics, such as having a large number of followers and discussing political topics more often. Therefore, we still anticipate that negativity will be shared more frequently among the majority of verified user types, especially news media and political figures who are more likely to engage in political discourse.

We tried to identify several major categories of verified users, including political figures, journalists, news organizations, entertainment, sports and organizations, and evaluated the tendency of their negative content to be shared by other users. To do so, employed three classification approaches in conjunction to evaluate these categories. First, we used manually curated dictionaries on the profile descriptions, which were informed by our topic modelling that provided us with a selection of frequent words that might make up one of these categories. For example, to identify political figures, we used partial matching to search for specific word parts such as "congress," "parliament," "senator," "governor," "mayor," and "council" (see Table S10 for all categories and corresponding word parts). Second, we leveraged to previous research on verified users, such as the lists created by Barberá (2015) as well as Rathje, Van Bavel, and van der Linden (2021) for political figures, and the documentation by Bellovary et al. (2021) for media outlets. We then linked the users identified as certain public figure types in these lists with our sample. Finally, we employed the tool "Demographer" which utilizes machine learning and natural language processing techniques to infer whether an account belongs to an individual or organization from multilingual social media data. Using these techniques, we were able to categorize around 46.96% of all verified users into accounting for 55.45% of the tweets in our dataset (see S11 Table below for exact breakdown).

**Table S10.** Word parts for each dictionary category. The table summarizes the word parts used for partial matching of public figures categories in the first step of categorization.

| Public Figure Category | Dictionary Word Parts |
| --- | --- |
| Entertainment | actor, actress, producer, writer, comedian, artist, music |
| Journalists | media, news, journal, reporter |
| Organisation | company, corporation, enterprise, group |
| Politics | congress, parliament, senator, governor, mayor, council, commissioner, activist |
| Sport | athlete, sport, player, referee |

**Table S11.** Breakdown of verified user types. The table summarizes the absolute count and percentage of tweets of users and by category, from our total sample of verified users before matching.

| Category | Users | Tweets | User % | Tweets % |
| --- | --- | --- | --- | --- |
| Entertainment | 4,281 | 202,767 | 9.24 | 11.24 |
| Journalists | 9,304 | 699,737 | 31.9 | 24.42 |
| News Outlets | 25 | 9,349 | 0.43 | 0.07 |
| Organisation | 2,249 | 203,063 | 9.26 | 5.9 |
| Politics | 751 | 22,892 | 1.04 | 1.97 |
| Sport | 1,284 | 78,287 | 3.57 | 3.37 |
| Other | 20,209 | 977,358 | 44.56 | 53.04 |

We in then tested whether some of these types of verified users drive the effect that negativity is shared more for verified users. To achieve this, we replicated the mixed model analysis, using the logarithmic value of the retweet count and the quadratic function of the continuous sentiment score from VADER as the predictors. In contrast to the previous analysis, where we added a simple binary variable for verified users, we added an interaction factor for the different user types (Ordinary User as well as the seven public figure types). We also included a random intercept for individual users to account for individual variability (see Table S12).

**Table S12.** Quadratic mixed model with the factors (Sentiment^1^, Sentiment^2^, Verified_user_types [7], Sentiment^1^ * Verified_user_types [7], Sentiment^2^ * Verified_user_types [7]) and number of retweets (log+1) as the dependent variable.

| **Fixed Effects** | | | | | |
| --- | --- | --- | --- | --- | --- |
|  | Estimate | *SE* | 95% CI | *t* | *p* |
| Intercept | 0.21 | 0.011 | 0.19 – 0.23 | 19.38 | <0.001 |
| Sentiment^1^ | 0.0048 | 0.003 | -0.0011 – 0.011 | 1.61 | 0.108 |
| Sentiment^2^ | 0.049 | 0.0056 | 0.038 – 0.060 | 8.74 | <0.001 |
| Entertainment | 0.41 | 0.018 | 0.38 – 0.45 | 22.96 | <0.001 |
| Journalists | 0.44 | 0.014 | 0.42 – 0.47 | 30.66 | <0.001 |
| News Outlets | 2.48 | 0.182 | 2.12 – 2.84 | 13.61 | <0.001 |
| Organization | 0.70 | 0.021 | 0.65 – 0.74 | 32.19 | <0.001 |
| Others | 0.69 | 0.013 | 0.66 – 0.71 | 53.37 | <0.001 |
| Politics | 0.93 | 0.029 | 0.88 – 0.99 | 31.45 | <0.001 |
| Sports | 0.46 | 0.028 | 0.41 – 0.52 | 16.60 | <0.001 |
| Sentiment^1^ × Entertainment | -0.14 | 0.0055 | -0.15 – -0.13 | -26.19 | <0.001 |
| Sentiment^2^ × Entertainment | 0.25 | 0.01 | 0.23 – 0.27 | 25.16 | <0.001 |
| Sentiment^1^ × Journalists | -0.066 | 0.0038 | -0.073 – -0.059 | -17.37 | <0.001 |
| Sentiment^2^ × Journalists | 0.098 | 0.0071 | 0.084 – 0.11 | 13.83 | <0.001 |
| Sentiment^1^ × News Outlets | -0.21 | 0.020 | -0.24 – -0.16 | -10.19 | <0.001 |
| Sentiment^2^ × News Outlets | 0.093 | 0.039 | 0.016 – 0.17 | 2.38 | 0.017 |
| Sentiment^1^ × Organization | -0.034 | 0.0053 | -0.045 – -0.023 | -6.37 | <0.001 |
| Sentiment^2^ × Organization | 0.043 | 0.0096 | 0.024 – 0.062 | 4.53 | <0.001 |
| Sentiment^1^ × Others | -0.13 | 0.0037 | -0.14 – -0.12 | -36.74 | <0.001 |
| Sentiment^2^ × Others | 0.20 | 0.0068 | 0.19 – 0.21 | 29.92 | <0.001 |
| Sentiment^1^ × Politics | -0.32 | 0.012 | -0.35 – -0.30 | -26.51 | <0.001 |
| Sentiment^2^ × Politics | 0.38 | 0.022 | 0.3360 – 0.4241 | 16.90 | <0.001 |
| Sentiment^1^ × Sports | -0.10 | 0.0087 | -0.12 – -0.087 | -11.95 | <0.001 |
| Sentiment^2^ × Sports | 0.25 | 0.016 | 0.22 – 0.28 | 16.21 | <0.001 |
| **Random Effects** | | | | | |
|  | |  | Variance | *SD* |  |
| User_id (Intercept) | |  | 0.90 | 0.95 |  |
| Residual | |  | 0.73 | 0.85 |  |
| **Model Fit** | | | | | |
| *R*^2^ | |  | Marginal | Conditional | |
|  | |  | 0.26 | 0.56 | |
| Model equation: log(retweets+1) ~ poly(compound,2, raw = TRUE) * scaled(Number_of_Followers) + (1 \| User) | | | | | |

Overall, the results of these additional analysis supported the idea that negativity is shared more for all types of public figures. In particular, political users and news medias’ negativity was shared significantly more than for other types of verified users, which seems consistent with the notion that negative content of users’ who produce more political content is shared more often.


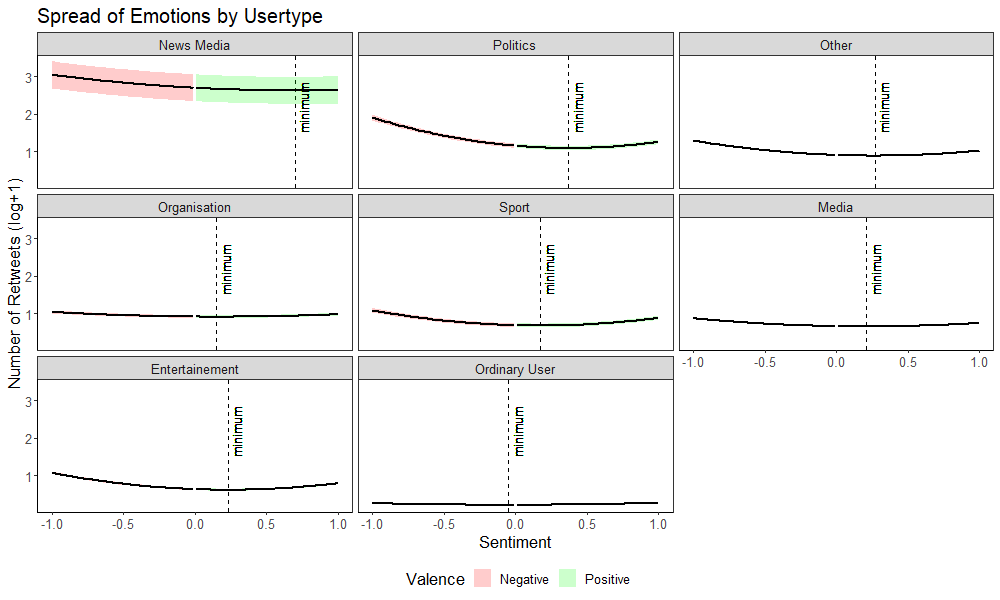


**Figure 9S.** Number of retweets as a function of sentiment and user type. The results suggest that stronger sentiment is associated with more retweets for all types of users. The local minimum for public figures is reached with a more positive emotional tweet, indicating that negativity is more strongly positively associated with number of retweets for all public figures than for ordinary users. Negativity increased shares most for political figures and news media.

# Average Emotion Expressed by Users

As outlined in the main manuscript, one notable difference between public figures and ordinary users was the prevalence of *positive language* in their posts. This finding may be attributed to the fact that social media is often used by individuals, including public figures, as a tool to enhance their public image (Citra, 2018). The larger a user’s network is, the more concerned they are about the impression they make (Hogan, 2010), which motivates them to express themselves more positively to avoid being perceived negatively (Buck, Losow, Murphy, & Costanzo, 1992; Lin, Tov, & Qiu, 2014). Public figures with their exceptionally large followings are therefore prone to generating more content with a positive sentiment. For users like public figures who primarily generate positive content, negativity could be shared more, as it is rarer and unexpected.

To test this idea, we calculated the average sentiment expressed by a user as the mean of their individual tweet sentiment. We then first tested the difference in emotion expressed between user types using this average sentiment score instead of using the categorical variables as in the main manuscript, before again testing the influence of these user characteristics on the likelihood that negativity is shared.

Replicating the previous analysis, public figures express themselves more positively (*b* = 0.064 [0.058, 0.069], SE = 0.0033, *t* (45911) = 19.00, *p* < .001, *R*^2^ = .012, see Figure S10) than ordinary users.


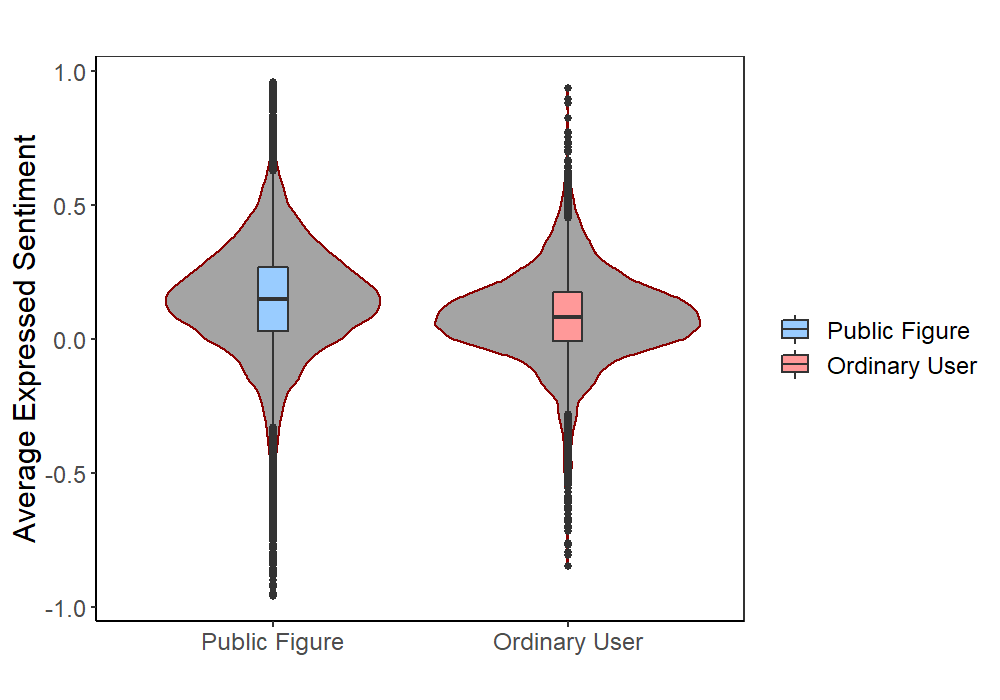


**Figure S10.** *Differences between public figures and ordinary users*. Results suggest that public figures express themselves using more positive language on average.

We again used a quadratic mixed model to predict the number of retweets using a quadratic function of the continuous sentiment score. We included the average sentiment expressed by a user as an interaction factor with sentiment in the model and added a random intercept for users to account for baseline differences in the average number of retweets that they get.

**Table S13.** Quadratic mixed model with five factors (Sentiment^2^, Sentiment^1^, Average_Sentiment_Expressed, Sentiment^1^ * Average_Sentiment_Expressed, Sentiment^2^ * Average_Sentiment_Expressed) and number of retweets (log+1) as the dependent variable.

| **Fixed Effects** | | | | | |
| --- | --- | --- | --- | --- | --- |
|  | Estimate | *SE* | 95% CI | *t* | *p* |
| Intercept | 0.46 | 0.0086 | 0.44 – 0.47 | 53.61 | .000 |
| Sentiment^1^ | -0.034 | 0.0022 | -0.038 – -0.029 | -15.50 | .000 |
| Sentiment^2^ | 0.16 | 0.0042 | 0.15 – 0.16 | 37.84 | .000 |
| Average_Sentiment_Expressed | 0.21 | 0.038 | 0.13 – 0.29 | 5.52 | .000 |
| Sentiment^1^ × Average_Sentiment_Expressed | -0.15 | 0.0014 | -0.17 – -0.11 | -10.47 | .000 |
| Sentiment^2^ × Average_Sentiment_Expressed | -0.081 | 0.0022 | -0.12 – -0.038 | -3.69 | .000 |
| **Random Effects** | | | | | |
|  | |  | Variance | *SD* |  |
| User_id (Intercept) | |  | 0.66 | 0.81 |  |
| Residual | |  | 0.46 | 0.68 |  |
| **Model Fit** | | | | | |
| *R*^2^ | |  | Marginal | Conditional | |
|  | |  | 0.0014 | 0.59 | |
| Model equation: log(retweets+1) ~ poly(compound,2, raw = TRUE) * scaled(Average_Sentiment_Expressed) + (1 \| User) | | | | | |

*Notes.* Model fit was calculated using the R package MuMIn (Barton & Barton, 2015) based on the paper of Nakagawa et al. (2017).


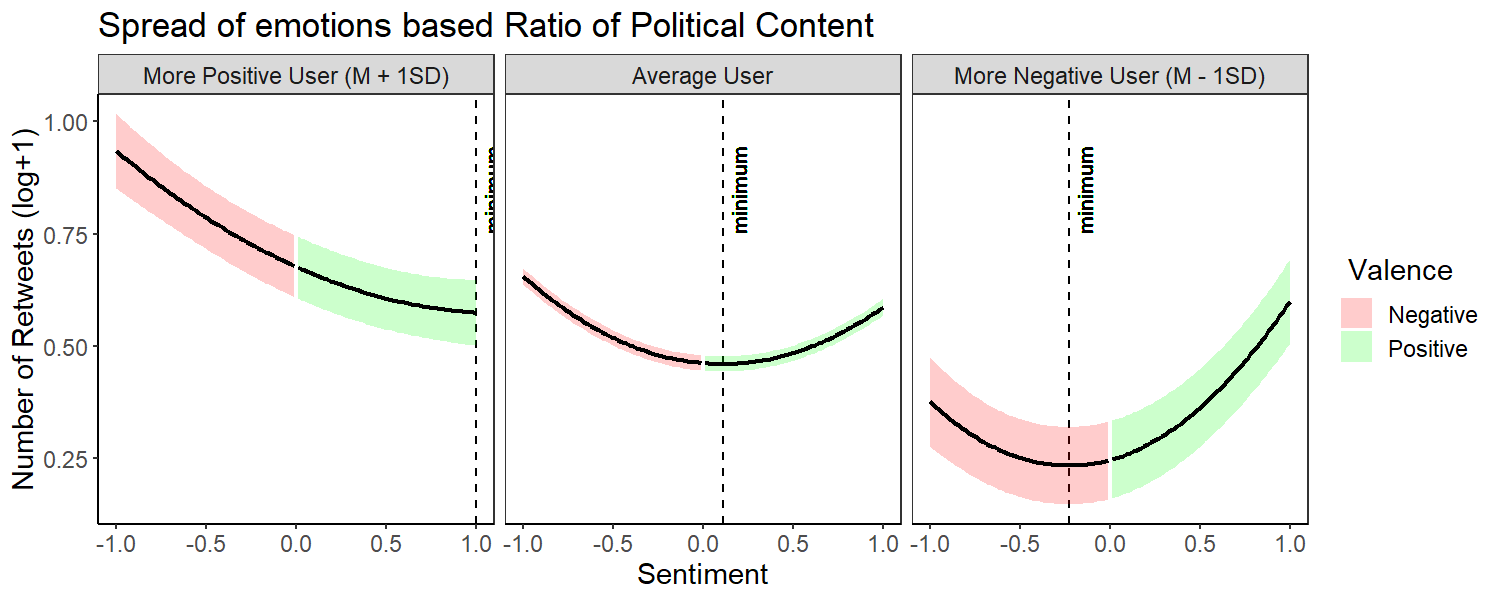


**Figure S11.** *The number of retweets as a function of sentiment and average sentiment expressed*. To visualize the interaction of two continuous variables (sentiment and average sentiment expressed), the panels show the predicted association between sentiment and the number of retweets at the mean sentiment expressed in the middle panel as well as the mean plus and minus one standard deviation (left and right panel respectively). The results indicate that tweets produced by users with more positive tweets on average show stronger associations between sentiment and content sharing and between negativity and content sharing.

# General Additive Model of Sentiment and Retweets for Public Figures and Ordinary Users

To verify whether quadratic models were the best choice, we conducted a general additive mixed model analysis using the mgcv package in R (Wood, 2015), on the full sample including 2,674,418 tweets of all users by 39,241 public figures and 6,677 ordinary users. Similar to the quadratic mixed model, we used the continuous VADER sentiment score to predict the log-modulus transformed retweet count. We chose four knots for the sentiment score, which determine the number of splines used for the fitting function. However, since general additive models do not allow for interactions of the splines, we conducted separate analyses for each user type. As use the full sample, that is not matched by their online activity, we included total tweets produced as a parametric covariate. Our findings confirmed the earlier observation that negativity spreads more widely than positivity. Furthermore, the results demonstrated that the quadratic function provided a good approximation of the relationship between sentiment and sharing.

## GAM for Public Figures.

**Table S14. GAM model:** General additive model with one smooth factors (sentiment score) using 4 knots and one parametric parameter (total number of tweets) predicting number of retweets as the dependent variable.

| Parametric Terms | | | | | |
| --- | --- | --- | --- | --- | --- |
|  | Estimate | *SE* | | *t* | *p* |
| Intercept | -0.098 | 0.0018 | | -53.38 | .000 |
| Total Tweets | 0.039 | 0.00031 | | 124.61 | .000 |
| Smooth-Terms | | | | | |
|  | edf | Ref.df | | *F* | *p* |
| Sentiment (4 knots) | 1.00 | 1.00 | | 1.25 | .884 |
| Random Effects | | | | | |
|  | |  | Variance | *SD* |  |
| Participant (Intercept) | |  | 0.017 | 0.13 |  |
| Residual | |  | 0.034 | 0.18 |  |
| Model fit | | | | | |
| *R*^2^ | |  |  | Adjusted | |
|  | |  |  | .14 | |
| Model equation: log(retweets+1) ~ (compound, knots = 4) + total_tweets + (1 \| User) | | | | | |

*Notes.* General additive mixed model was computed via mgcv package in R (Wood, 2015).


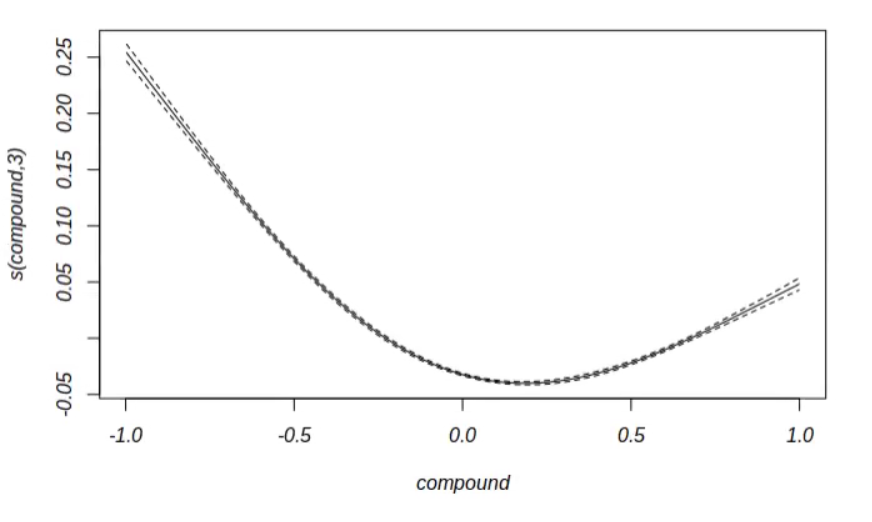


**Figure S12.** Results of sentiment predicting number of retweets for the content produced by public figures. We were able to replicate the previous finding, that negativity is shared more for public figures using GAM, which allows the function to be fitted more flexible to the data (4 knots). After visual inspection, we concluded that a quadratic function is indeed an appropriate approximation of the relationship between sentiment and retweets.

## GAM for Ordinary Users

**Table S15. GAM model:** General additive model with one smooth factors (sentiment score) using 4 knots and one parametric parameter (total number of tweets) predicting number of retweets as the dependent variable.

| Parametric Terms | | | | | |
| --- | --- | --- | --- | --- | --- |
|  | Estimate | *SE* | | *t* | *p* |
| Intercept | 0.15 | 0.0047 | | 32.52 | .000 |
| Total Tweets | -0.00018 | 0.000036 | | -5.20 | .000 |
| Smooth-Terms | | | | | |
|  | edf | Ref.df | | *F* | *p* |
| Sentiment (4 knots) | 2.98 | 2.98 | | 133.2 | .002 |
| Random Effects | | | | | |
|  | |  | Variance | *SD* |  |
| Participant (Intercept) | |  | 0.092 | 0.30 |  |
| Residual | |  | 0.17 | 0.41 |  |
| Model fit | | | | | |
| *R*^2^ | |  |  | Adjusted | |
|  | |  |  | .09 | |
| Model equation: log(retweets+1) ~ (compound, knots = 4) + total_tweets + (1 \| User) | | | | | |

*Notes.* General additive mixed model was computed via mgcv package in R (Wood, 2015).


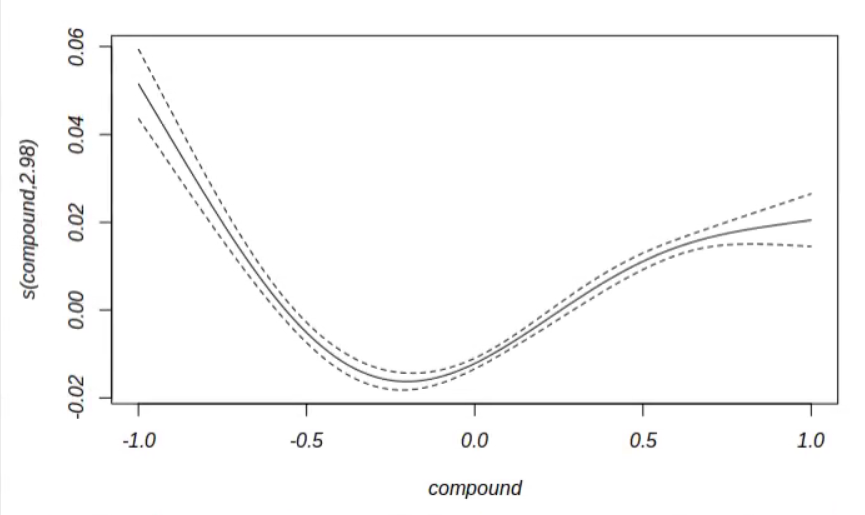


**Figure S13.** Results of sentiment predicting number of retweets for the content produced by Ordinary Users. We were able to replicate the previous finding, that both positivity and negativity is associated with increased shares using GAM, which allows the function to be fitted more flexible to the data (4 knots). After visual inspection, we concluded that a quadratic function is indeed an appropriate approximation of the relationship between sentiment and retweets.

# References

Barton, K., & Barton, M. K. (2015). Package ‘MuMIn’. *Version, 1, 18*.

Buck, R., Losow, J. I., Murphy, M. M., & Costanzo, P. (1992). Social facilitation and inhibition of emotional expression and communication. *Journal of Personality and Social Psychology, 63*(6), 962.

Citra, A. (2018). Maintaining the Good Image of a Public Figure through the use of Reputation Marketing. *Expose: Jurnal Ilmu Komunikasi, 1*(1).

Hayes, A. F. (2017). *Introduction to mediation, moderation, and conditional process analysis: A regression-based approach*: Guilford publications.

Hogan, B. (2010). The presentation of self in the age of social media: Distinguishing performances and exhibitions online. *Bulletin of Science, Technology & Society, 30*(6), 377-386.

Lin, H., Tov, W., & Qiu, L. (2014). Emotional disclosure on social networking sites: The role of network structure and psychological needs. *Computers in Human Behavior, 41*, 342-350.

Nakagawa, S., Johnson, P. C., & Schielzeth, H. (2017). The coefficient of determination R 2 and intra-class correlation coefficient from generalized linear mixed-effects models revisited and expanded. *Journal of the Royal Society Interface, 14*(134), 20170213.

Thelwall, M., Buckley, K., & Paltoglou, G. (2012). Sentiment strength detection for the social web. Journal of the American Society for Information Science and Technology, 63(1), 163-173
